# Effect of Perioperative Dexmedetomidine on Delayed Graft Function Following a Donation-After-Cardiac-Death Kidney Transplant: A Randomized Clinical Trial

**DOI:** 10.1001/jamanetworkopen.2022.15217

**Published:** 2022-06-03

**Authors:** Xi-sheng Shan, Lin-kun Hu, Yiqing Wang, Hua-yue Liu, Jun Chen, Xiao-wen Meng, Jin-xian Pu, Yu-hua Huang, Jian-quan Hou, Xiao-mei Feng, Hong Liu, Lingzhong Meng, Ke Peng, Fu-hai Ji

**Affiliations:** 1 Department of Anesthesiology, The First Affiliated Hospital of Soochow University, Suzhou, Jiangsu, China; 2 Institute of Anesthesiology, Soochow University, Suzhou, Jiangsu, China; 3 Department of Urology, The First Affiliated Hospital of Soochow University, Suzhou, Jiangsu, China; 4 Department of Neurology, The First Affiliated Hospital of Soochow University, Suzhou, Jiangsu, China; 5 Department of Anesthesiology, University of Utah Health, Salt Lake City; 6 Department of Anesthesiology and Pain Medicine, University of California, Davis Health, Sacramento; 7 Department of Anesthesiology, The First Affiliated Hospital, Zhejiang University School of Medicine, Hangzhou, Zhejiang, China

## Abstract

**Importance:**

Delayed graft function (DGF) is a risk factor for acute rejection and graft failure after kidney transplant. Previous studies have suggested that dexmedetomidine may be renoprotective, but whether the use of dexmedetomidine would improve kidney allograft function is unknown.

**Objective:**

To investigate the effects of perioperative dexmedetomidine on DGF following a donation-after-cardiac-death (DCD) kidney transplant.

**Design, Setting, and Participants:**

This single-center, double-blind, placebo-controlled randomized clinical trial was conducted at The First Affiliated Hospital of Soochow University in Suzhou, China. Adults (18 years or older) who were scheduled for DCD kidney transplant were enrolled between September 1, 2019, and January 28, 2021, and then randomized to receive either dexmedetomidine or normal saline (placebo). One-year postoperative outcomes were recorded. All analyses were based on the modified intention-to-treat population.

**Interventions:**

Patients who were randomized to the dexmedetomidine group received a 24-hour perioperative dexmedetomidine intravenous infusion (0.4 μg/kg/h intraoperatively and 0.1 μg/kg/h postoperatively). Patients who were randomized to the normal saline group received an intravenous infusion of the placebo with the same dose regimen as the dexmedetomidine.

**Main Outcomes and Measures:**

The primary outcome was the incidence of DGF, defined as the need for dialysis in the first posttransplant week. The prespecified secondary outcomes were in-hospital repeated dialysis in the first posttransplant week, in-hospital acute rejection, and serum creatinine, serum cystatin C, estimated glomerular filtration rate, need for dialysis, and patient survival on posttransplant day 30.

**Results:**

Of the 114 patients enrolled, 111 completed the study (mean [SD] age, 43.4 [10.8] years; 64 male patients [57.7%]), of whom 56 were randomized to the dexmedetomidine group and 55 to the normal saline group. Dexmedetomidine infusion compared with normal saline reduced the incidence of DGF (17.9% vs 34.5%; odds ratio [OR], 0.41; 95% CI, 0.17-0.98; *P* = .04) and repeated dialysis (12.5% vs 30.9%; OR, 0.32; 95% CI, 0.13-0.88; *P* = .02, which was not statistically significant after multiple testing corrections), without significant effect on other secondary outcomes. Dexmedetomidine vs normal saline infusion led to a higher median (IQR) creatinine clearance rate on postoperative days 1 (9.9 [4.9-21.2] mL/min vs 7.9 [2.0-10.4] mL/min) and 2 (29.6 [9.7-67.4] mL/min vs 14.6 [3.8-45.1] mL/min) as well as increased median (IQR) urine output on postoperative days 2 (106.5 [66.3-175.6] mL/h vs 82.9 [27.1-141.9] mL/h) and 7 (126.1 [98.0-151.3] mL/h vs 107.0 [82.5-137.5] mL/h) and at hospital discharge discharge (110.4 [92.8-121.9] mL/h vs 97.1 [77.5-113.8] mL/h). Three patients (5.5%) from the normal saline group developed allograft failure by the post hoc 1-year follow-up visit.

**Conclusions and Relevance:**

This randomized clinical trial found that 24-hour perioperative dexmedetomidine decreased the incidence of DGF after DCD kidney transplant. The findings support the use of dexmedetomidine in kidney transplants.

**Trial Registration:**

Chinese Clinical Trial Registry Identifier: ChiCTR1900025493

## Key Points

**Question:**

Does perioperative use of dexmedetomidine reduce delayed graft function following a donation-after-cardiac-death (DCD) kidney transplant?

**Findings:**

In this randomized clinical trial of 111 adults who underwent a DCD kidney transplant, delayed graft function occurred in 17.9% of patients who received the 24-hour perioperative dexmedetomidine infusion, which was significantly lower than the 34.5% occurrence in patients who received normal saline.

**Meaning:**

The findings of this trial support the perioperative use of dexmedetomidine for reducing delayed graft function in DCD kidney transplants.

## Teaser

This randomized clinical trial compares outcomes among patients who received a donated-after-cardiac-death kidney allograft and either dexmedetomidine or placebo during and after the surgical procedure.

## Introduction

The point prevalence of end-stage kidney disease in the US increased from 727 per million in 1990 to 2206 per million in 2016.^1^ Kidney transplant is an established effective treatment for end-stage kidney disease,^2,3^ with the number of US patients who received kidney allografts increasing from 10 011 in 1991 to 19 355 in 2016.^1^ However, various complications can emerge during the posttransplant course, such as delayed graft function (DGF).^4,5^ The incidence of DGF is about 4% to 10% in living-donor kidney transplant and 20% to 50% in deceased-donor kidney transplant.^6^ Delayed graft function has been associated with ischemia-reperfusion injury^7^ as well as a higher risk of acute rejection and reduced long-term allograft survival.^6,8^

Dexmedetomidine is a selective α_2_-adrenoreceptor agonist with sedative, anxiolytic, sympatholytic, and analgesic effects.^9^ Dexmedetomidine may be renoprotective, which is likely associated with the attenuation of ischemia-reperfusion injury.^10,11^ A recent meta-analysis suggested an association between perioperative dexmedetomidine and reduced acute kidney injury after cardiac surgery.^12^ A retrospective cohort study suggested that dexmedetomidine use was associated with decreased incidence of DGF after isolated kidney transplant or multiorgan transplant.^13^ However, to date, no randomized clinical trial has investigated the effect of dexmedetomidine on kidney allograft function.

In this single-center, double-blind, placebo-controlled randomized clinical trial, we investigated the effects of perioperative dexmedetomidine on DGF following a donation-after-cardiac-death (DCD) kidney transplant. We hypothesized that perioperative dexmedetomidine infusion reduces the incidence of DGF after DCD kidney transplant. We compared the incidence of DGF between patients with kidney allograft who received dexmedetomidine and patients who received normal saline (placebo) during and after surgery for a total of 24 hours.

## Methods

This randomized clinical trial was conducted at The First Affiliated Hospital of Soochow University in Suzhou, China. The trial was approved by the ethics committee of The First Affiliated Hospital of Soochow University. Written informed consent was obtained from all patients. We followed the Consolidated Standards of Reporting Trials (CONSORT) reporting guideline. The trial protocol and statistical plan are provided in Supplement 1.

### Organ Donation and Procurement

The procedures of organ donation and transplant conformed to the National Guidelines for Donation after Cardiac Death in China.^14^ All of the donors were controlled donors after cardiac death.^15,16^ Apnea test was performed to determine donor suitability for DCD.^17,18^ Details of organ donation and procurement are presented in the eMethods in Supplement 2.

### Donor Data Collection and Risk Assessment

Donor data were obtained from the Organ Procurement Organization records. The time between withdrawal of life-sustaining treatment and asystole, asystolic warm ischemic time (defined as the interval from asystole to the start of cold preservation),^18^ and cold ischemic time (defined as the interval from the start of cold preservation to the start of graft reperfusion) were collected.^19^ Donor kidneys were assessed for the expanded-criteria donor^20^ subgroup and evaluated using the donor-only US Kidney Donor Risk Index (KDRI) and the Chinese-donor DGF risk prediction model (eMethods in Supplement 2).^21,22,23^

### Patients, Randomization, and Blinding

Patients who were 18 years or older, diagnosed with end-stage kidney disease, undergoing kidney replacement therapy, and scheduled for DCD kidney transplant were eligible for inclusion. Patients who had sick sinus syndrome, an atrioventricular block, a left ventricular ejection fraction less than 30%, or a multiorgan transplant were excluded.

All of the patients were of Han Chinese ethnicity. Race and ethnicity data were not collected because, we believe, they would have had no impact on the perioperative care and study outcomes.

**Figure 1.**
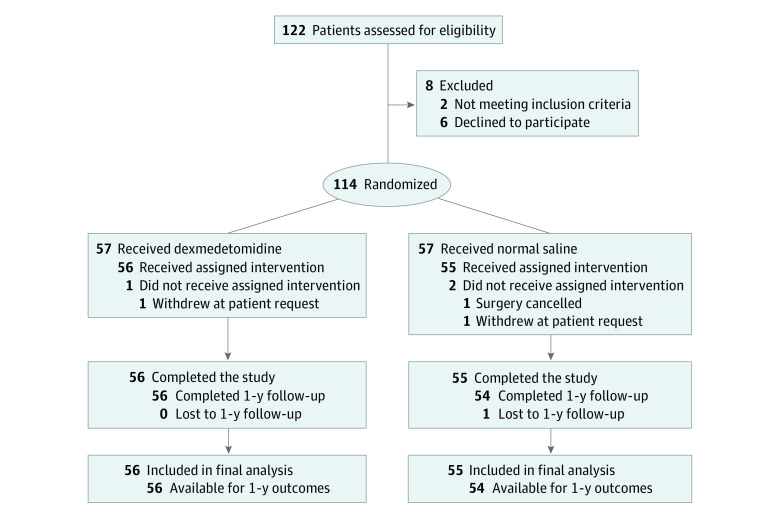
CONSORT Flow Diagram

Eligible adults were enrolled from September 1, 2019, to January 28, 2021. Patients were randomized to either dexmedetomidine or normal saline (Figure 1) using a 1:1 ratio and permuted block sizes of 2 and 4. Randomization was concealed using identical opaque envelopes that were sealed and stored in a locked cabinet. An independent research nurse prepared the medications according to the randomization results. Dexmedetomidine and normal saline were each kept in syringes that were labeled only with the patient number. There was no way to distinguish the contents of the syringes because both dexmedetomidine and saline are colorless and the syringes were identical. The patients, clinicians, and outcome assessors were all blinded to the randomization.

### Anesthesia and Dialysis Treatment

Intraoperative monitoring included noninvasive cuff blood pressure, electrocardiography, pulse oximetry, end-tidal carbon dioxide, radial artery blood pressure, central venous pressure, and bispectral index. Intravenous infusion of lactated Ringer solution was provided. After anesthesia induction, patients were endotracheally intubated and mechanically ventilated. Anesthesia was maintained using sevoflurane titrated to bispectral index values of 40 to 60. Hypotension (mean arterial pressure <65 mm Hg or a decrease of ≥20% from baseline) and bradycardia (heart rate <50 beats/min) were treated. A sufentanil-based patient-controlled analgesia was used for postoperative pain relief. Additional information on anesthetic care is available in the eMethods in Supplement 2.

The perioperative care of patients who underwent kidney transplant was based on the Kidney Disease: Improving Global Outcomes guideline.^4,24^ The perioperative dialysis treatment is presented in the eMethods in Supplement 2.

### Interventions and Outcomes

The dexmedetomidine group received an intravenous infusion of dexmedetomidine, 0.4 μg/kg/h, immediately after anesthesia induction and continued throughout the procedure. After surgery, all patients were transferred to the designated transplant unit and received dexmedetomidine, 0.1 μg/kg/h. Dexmedetomidine was administered for a total of 24 hours. The dose regimen of dexmedetomidine was based on the dose used in previous studies in cardiac surgery^25^ and older patients who underwent noncardiac surgery.^26^ The control group received an intravenous infusion of normal saline administered in the same dose regimen as dexmedetomidine.

The primary outcome was the incidence of DGF, defined as the need for dialysis during the first posttransplant week.^4,5^ The prespecified secondary outcomes were in-hospital repeated dialysis during the first posttransplant week; in-hospital acute rejection; and serum creatinine, serum cystatin C, estimated glomerular filtration rate (eGFR), need for dialysis, and patient survival on posttransplant day 30. Repeated dialysis was defined as 2 or more dialysis sessions during the first posttransplant week.^27^ Acute rejection was confirmed by biopsy of the kidney allograft. The eGFR was calculated using the Chronic Kidney Disease Epidemiology Collaboration equation.^28^

Several in-hospital and long-term outcomes were assessed post hoc: (1) creatinine reduction ratio on posttransplant day 2^29^; (2) proportion of creatinine reduction ratio on posttransplant day 2 that was less than 30%^30^; (3) number of dialysis treatments in the first posttransplant week; (4) timing of acute rejection diagnosis; (5) acute rejection in the first posttransplant week; (6) pneumonia; (7) deep vein thrombosis; (8) incidence of DGF in donor criteria subgroups and KDRI subgroups; and (9) serum creatinine, serum cystatin C, eGFR, acute rejection, allograft failure (defined as the return to regular dialysis, graft removal, or patient death),^6,31^ and patient survival at 1 posttransplant year.

### Perioperative Data

The perioperative data were (1) graft function–related parameters: baseline serum creatinine and serum cystatin C before surgery as well as serum creatinine, serum cystatin C, creatinine clearance rate, and urine output on postoperative days (PODs) 1, 2, 3, 5, and 7, and at the time of hospital discharge; (2) arterial blood gas and electrolytes at the end of surgery; (3) visual analog scale pain score (range: 0-10, with 0 indicating no pain and 10 indicating most severe pain) at 30 minutes, 24 hours, and 48 hours postoperatively; (4) sufentanil consumption over 24 hours and 48 hours postoperatively; (5) perioperative bradycardia and hypotension; (6) transplant induction therapy and immunosuppressive medications; (7) duration of surgery, anastomosis time, time to extubation, and length of hospital stay; (8) intraoperative infusion of lactated Ringer solution; (9) furosemide use in the first posttransplant week; (10) level of postoperative nursing care (level I indicating intensive care, and level II indicating conventional care) on PODs 1 to 3; and (11) level of physical activity assessed using the Barthel index score (range, 0-100, with lower scores indicating increased disability)^32,33^ on PODs 1 to 3.

The creatinine clearance rate, urine output, and immunosuppressive medications are presented in the eMethods in Supplement 2. A single multidisciplinary team provided perioperative care to ensure consistency and efficiency. Adherence by clinicians and outcome assessors to the study protocol was achieved by training the research personnel and reviewing the case report forms.

### Statistical Analysis

According to the literature, the incidence of DGF after DCD kidney transplant was 45% to 55%.^31^ The (unpublished) pilot study we conducted showed that 9 of 20 patients (45%) who did not receive dexmedetomidine experienced DGF, which was in line with the previous report. The therapeutic effect of dexmedetomidine on DGF is unknown. We hypothesized that dexmedetomidine would reduce the incidence of DGF by 50%. Therefore, this trial required 54 patients in each group with a power of 80% at a significance of α = .05. We decided to recruit 114 patients (with 57 in each group) with consideration of a possible dropout rate of 5%.

Continuous variables were presented as means (SDs) or medians (IQRs), depending on data distribution. The categorical variables were presented as numbers (percentages). The between-group difference in the DGF incidence was analyzed using the χ^2^ test, and the therapeutic effect was assessed with odds ratios (ORs) and 95% CIs. A 2-sided *P* < .05 indicated a statistically significant difference. As appropriate, the secondary outcomes were analyzed with the unpaired, 2-tailed *t* test; Mann-Whitney rank sum test; χ^2^ test; or Fisher exact test. The therapeutic effect was assessed using the OR (or difference) and 95% CI. Multiple testing was corrected using the Bonferroni method, with *P* < .007 regarded as statistically significant. Because multiple testing corrections were not planned for the nonoutcome perioperative data and post hoc analyses, these results should be considered exploratory.^34^ The effects of interventions on DGF and 1-year postoperative acute rejection, allograft failure, and patient survival were assessed using the Kaplan-Meier curve and the log-rank test, and the therapeutic effect was analyzed with the hazard ratio (HR) and 95% CI.

All analyses were based on the modified intention-to-treat population principle, which included any randomized patient who had undergone kidney transplant and for whom the result of the primary outcome was available. Neither an interim analysis nor missing data imputation was planned a priori. Statistical analyses were performed using the SPSS software, version 23.0 (IBM SPSS).

## Results

Of the 122 patients screened for eligibility, 8 were excluded and 114 were randomized (Figure 1). One patient did not undergo the planned transplant because of intraabdominal infection, and 2 patients withdrew their informed consent. The remaining 111 patients (56 in the dexmedetomidine group, and 55 in the normal saline group) underwent the scheduled transplant and had available primary outcome data. One patient in the normal saline group was lost to the 1-year postoperative follow-up.

In total, 47 (42.3%) female and 64 (57.7%) male individuals with a mean (SD) age of 43.4 (10.8) years participated in the trial (Table 1). The donors had a mean (SD) age of 37.4 (13.9) years. Approximately 80% of donors after cardiac death became unstable during the 10-minute apnea test. The panel reactive antibody was less than 10% for all patients. No patients had previous transplants or a preemptive transplant. The median (IQR) time between treatment withdrawal and asystole was 8.0 (6.0-11.8) minutes and 9.0 (7.0-13.0) minutes (ranging from 4 to 48 minutes), and the median (IQR) asystolic warm ischemic time was 9.5 (8.0-10.0) minutes and 10.0 (6.0-11.0) minutes, in the dexmedetomidine and normal saline groups, respectively.

**Table 1.** Baseline Characteristics

	Patients, No. (%)	
	Dexmedetomidine group (n = 56)	Normal saline group (n = 55)
**Patient characteristics**		
Age, mean (SD), y	43.5 (10.7)	43.3 (10.9)
Sex		
Female	20 (35.7)	27 (49.1)
Male	36 (64.3)	28 (50.9)
BMI, mean (SD)	21.8 (3.2)	21.1 (3.2)
Comorbidity		
Hypertension	56 (100)	55 (100)
Diabetes	3 (5.4)	3 (5.5)
Obesity (BMI >30)	1 (1.8)	0
COPD	3 (5.4)	2 (3.6)
Cause of end-stage kidney disease		
Glomerulonephritis	12 (21.4)	14 (25.5)
Nephrotic syndrome	8 (14.3)	9 (16.4)
Polycystic kidney disease	1 (1.8)	1 (1.8)
Other	35 (62.5)	31 (56.4)
Kidney replacement therapy before transplant		
Hemodialysis	34 (60.7)	38 (69.1)
Peritoneal dialysis	22 (39.3)	17 (30.9)
Length of dialysis, median (IQR), mo	22.5 (10.3-36.8)	24.0 (12.0-57.0)
ABO blood type		
A	15 (26.8)	17 (30.9)
B	13 (23.2)	13 (23.6)
AB	9 (16.1)	8 (14.5)
O	19 (33.9)	17 (30.9)
Baseline hemodynamics		
Blood pressure, mean (SD), mm Hg	117.1 (20.1)	120.4 (20.8)
Heart rate, mean (SD), bpm	82.3 (13.1)	80.5 (12.9)
Baseline serum creatinine, mean (SD), mg/dL	10.52 (3.00)	10.28 (3.41)
**Donor characteristics**		
Age, mean (SD), y	38.1 (12.6)	36.7 (15.2)
Sex		
Female	13 (23.2)	13 (23.6)
Male	43 (76.8)	42 (76.4)
BMI, mean (SD)	23.8 (2.7)	23.4 (3.1)
Comorbidity		
Hypertension	18 (32.1)	15 (27.3)
Diabetes	3 (5.4)	2 (3.6)
Obesity (BMI >30)	0	0
ABO blood type		
A	15 (26.8)	17 (30.9)
B	13 (23.2)	13 (23.6)
AB	9 (16.1)	8 (14.5)
O	19 (33.9)	17 (30.9)
Primary cause of death		
Traumatic brain injury	38 (67.9)	35 (63.6)
Cerebral hemorrhage	14 (25.0)	15 (27.3)
Other	4 (7.1)	5 (9.1)
Last serum creatinine, median (IQR), mg/dL	0.98 (0.66-1.51)	1.00 (0.64-1.55)
Last serum NGAL, median (IQR), ng/mL	364.0 (170.2-672.6) [n = 53]	329.0 (174.0-552.0) [n = 49]
Expanded-criteria donor	4 (7.1)	4 (7.3)
Apnea test <10 min	45 (80.4)	44 (80.0)
Donor-only US KDRI^a^		
Scores, mean (SD)	1.20 (0.31)	1.22 (0.31)
Quintile		
1 (0.45-0.78)	2 (3.6)	1 (1.8)
2 (0.79-0.95)	11 (19.6)	10 (18.2)
3 (0.96-1.14)	13 (23.2)	12 (21.8)
4 (1.15-1.44)	20 (35.7)	22 (40.0)
5 (≥1.45)	10 (17.9)	10 (18.2)
Chinese-donor DGF risk prediction model^b^		
Scores, median (IQR)	7 (1-11)	7 (2-11)
Quartile		
1 (0-9)	39 (69.6)	38 (69.1)
2 (10-19)	15 (26.8)	15 (27.3)
3 (20-29)	2 (3.6)	2 (3.6)
4 (≥30)	0	0
**Other characteristics**		
Human leukocyte antigen mismatch, median (IQR)	5 (4-6)	5 (4-5)
Panel reactive antibody <10%	56 (100)	55 (100)
Time between withdrawal and asystole, median (IQR), min	8.0 (6.0-11.8)	9.0 (7.0-13.0)
Asystolic warm ischemic time, median (IQR), min	9.5 (8.0-10.0)	10.0 (6.0-11.0)
Cold ischemic time, median (IQR), h	9.0 (6.0-15.0)	9.0 (6.0-14.0)

Abbreviations: BMI, body mass index (calculated as weight in kilograms divided by height in meters squared); bpm, beats per minute; COPD, chronic obstructive pulmonary disease; DGF, delayed graft function; KDRI, Kidney Donor Risk Index; NGAL, neutrophil gelatinase–associated lipocalin.

SI conversion factor: To convert serum creatinine levels to micromole per liter, multiply by 88.4.

^a^ A higher quintile in the donor-only US KDRI indicates a lower rate of long-term graft survival.

^b^ A higher quartile in the Chinese-donor DGF risk prediction model indicates a higher risk of DGF.

The blood gas and electrolyte parameters at the end of surgery were within the normal ranges in both groups (Table 2). The 2 groups had comparable incidences of bradycardia and hypotension, induction therapy, immunosuppressive medications, intraoperative fluid infusion, posttransplant use of furosemide, postoperative nursing care, and Barthel index scores. The mean (SD) anastomosis time was 37.2 (10.0) minutes in the dexmedetomidine group and 38.2 (11.0) minutes in the normal saline group. The median length of hospital stay was 25 days in both groups.

**Table 2.** Perioperative Data

Variable	Median (IQR)			*P* value
	Dexmedetomidine group (n = 56)	Normal saline group (n = 55)	Difference or OR (95% CI)	
Postoperative blood gas and electrolytes levels				
pH, mean (SD)	7.37 (0.07)	7.38 (0.06)	Difference, −0.01 (−0.04 to 0.01)	.39
Pco_2_, mm Hg	37.9 (35.9 to 40.9)	37.0 (34.0 to 40.0)	Difference, 0.9 (−0.5 to 2.6)	.17
Po_2_, mean (SD), mm Hg	256.8 (70.2)	271.0 (68.6)	Difference, −14.2 (−40.3 to 11.9)	.28
Hemoglobin, mean (SD), g/dL	10.4 (1.5)	10.2 (1.8)	Difference, 0.2 (−0.4 to 0.8)	.53
Potassium, mean (SD), mEq/L	4.8 (1.0)	4.6 (0.8)	Difference, 0.2 (−0.2 to 0.5)	.28
Sodium, mean (SD), mEq/L	138.3 (2.2)	138.6 (2.5)	Difference, 0.3 (−1.2 to 0.6)	.48
Bicarbonate, mean (SD), mEq/L	22.8 (2.2)	22.4 (2.0)	Difference, 0.4 (−0.4 to 1.2)	.33
Lactic acid, mmol/L	1.0 (0.8 to 1.1)	1.0 (0.7 to 1.3)	Difference, 0 (−0.1 to 0.2)	.79
Postoperative pain and analgesic consumption				
VAS pain scores^a^				
at 30 min	2 (2 to 3)	3 (2 to 4)	Difference, −1 (−1 to 0)	.004
at 24 h	3 (2 to 3)	3 (3 to 4)	Difference, 0 (−1 to 0)	.001
at 48 h	3 (3 to 3)	3 (3 to 3)	Difference, 0 (0 to 0)	.15
Sufentanil consumption, μg				
0-24 h	48 (45 to 50)	49 (46 to 50)	Difference, −1 (−2 to 0)	.32
0-48 h	96 (94 to 98)	96 (94 to 98)	Difference, 0 (−1 to 1)	.97
Perioperative hemodynamic event				
Bradycardia, No. (%)	9 (16.1)	5 (9.1)	OR, 1.92 (0.59 to 5.39)	.27
Hypotension, No. (%)	8 (14.3)	6 (10.9)	OR, 1.36 (0.41 to 4.38)	.59
Induction therapy				
Anti-CD25, No. (%)	41 (73.2)	39 (70.9)	OR, 1.12 (0.50 to 2.57)	.79
Antithymocyte globulin, No. (%)	15 (26.8)	16 (29.1)	OR, 0.89 (0.39 to 2.02)	.79
Immunosuppressive medication				
Tacrolimus, No. (%)	52 (92.9)	50 (90.9)	OR, 1.30 (0.33 to 5.12)	.74
Cyclosporine, No. (%)	4 (7.1)	5 (9.1)	OR, 0.77 (0.20 to 3.03)	.74
Mycophenolate mofetil, No. (%)	37 (66.1)	38 (69.1)	OR, 0.87 (0.41 to 2.00)	.73
Mycophenolic acid, No. (%)	19 (33.9)	17 (30.9)	OR, 1.15 (0.50 to 2.44)	.73
Methylprednisolone, mg	480 (423 to 480)	480 (400 to 480)	Difference, 0 (0 to 0)	.14
Intraoperative fluid infusion, mL	1100 (895 to 1300)	1000 (900 to 1250)	Difference, 100 (−100 to 110)	.90
Posttransplant furosemide use, No. (%)	33 (58.9)	38 (69.1)	OR, 0.64 (0.29 to 1.40)	.26
Furosemide dose in first posttransplant week, mean (SD), mg	164.2 (92.6) [n = 33]	165.8 (74.1) [n = 38]	Difference, −1.5 (−41.7 to 38.7)	.94
Level of postoperative nursing care (I or II), No. level I/No. level II^b^				
POD 1	56/0	55/0	NA	>.99
POD 2	56/0	55/0	NA	>.99
POD 3	4/52	5/50	OR, 0.77 (0.23 to 2.79)	.74
Level of physical activity (Barthel index score)^c^				
POD 1	25 (25 to 30)	30 (25 to 30)	OR, −5 (−5 to 0)	.48
POD 2	40 (31 to 50)	40 (30 to 45)	OR, 0 (0 to 5)	.32
POD 3	85 (75 to 85)	85 (75 to 85)	OR, 0 (0 to 0)	.63
Duration of surgery, min	180 (155 to 200)	185 (160 to 210)	Difference, −5 (−20 to 5)	.24
Anastomosis time, mean (SD), min	37.2 (10.0)	38.2 (11.0)	Difference, −1.0 (−4.9 to 3.0)	.63
Time to extubation, min	16 (13 to 25)	18 (15 to 22)	Difference, −2 (−3 to 3)	.82
Length of hospital stay, d	25 (22 to 28)	25 (22 to 29)	Difference, 0 (−2 to 1)	.74

Abbreviations: NA, not applicable; OR, odds ratio; POD, postoperative day; VAS, visual analog scale.

SI conversion factors: To convert Pco_2_ and Po_2_ levels to kilopascal, multiply by 0.133; hemoglobin level to gram per liter, multiply by 10.0; potassium, sodium, and bicarbonate levels to millimoles per liter, multiply by 1.0.

^a^ VAS pain score range: 0 to 10, with 0 indicating no pain and 10 indicating most severe pain.

^b^ Level of postoperative nursing care: I indicating intensive care and II indicating conventional care.

^c^ Barthel index score range: 0 to 100, with lower scores indicating increased disability.

**Figure 2.**
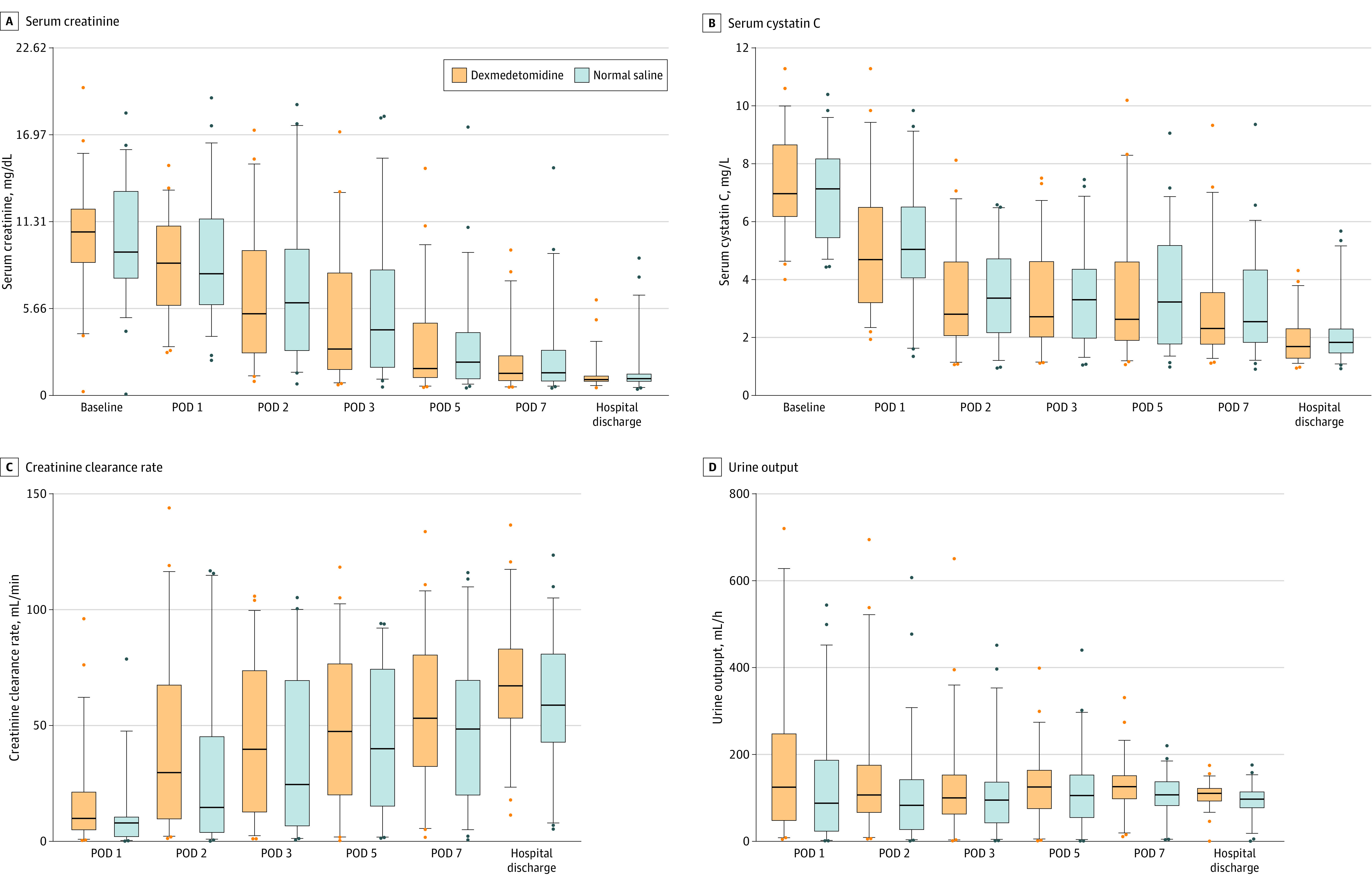
Serial Changes in Graft Function Parameters During the Hospitalization Period Box indicates IQR; dots, outliers; line within the box, median data; whiskers, 5th and 95th percentile values; POD, postoperative day. SI conversion factor: To convert serum creatinine levels to micromole per liter, multiply by 88.4.

The graft function–related results are shown in Figure 2 and eTable in Supplement 2. The median (IQR) creatinine clearance rate was higher in the dexmedetomidine group than in the normal saline group on POD 1 (9.9 [4.9-21.2] mL/min vs 7.9 [2.0-10.4] mL/min; difference, 2.0 [95% CI, 0.5-6.8] mL/min) and POD 2 (29.6 [9.7-67.4] mL/min vs 14.6 [3.8-45.1] mL/min; difference, 15.0 [95% CI, 0.4-18.5] mL/min). In addition, the dexmedetomidine group compared with the normal saline group had higher median (IQR) urine output on POD 2 (106.5 [66.3-175.6] mL/h vs 82.9 [27.1-141.9] mL/h; difference, 23.6 [95% CI, 0.2-59.0] mL/h), POD 7 (126.1 [98.0-151.3] mL/h vs 107.0 [82.5-137.5] mL/h; difference, 19.1 [95% CI, 1.7- 36.3] mL/h), and hospital discharge (110.4 [92.8-121.9] mL/h vs 97.1 [77.5-113.8] mL/h; difference, 13.3 [95% CI, 4.2-22.5] mL/h).

### Primary Outcome

Delayed graft function occurred in 10 of 56 patients (17.9%) in the dexmedetomidine group and 19 of 55 patients (34.5%) in the normal saline group (OR, 0.41; 95% CI, 0.17-0.98; *P* = .04) (Table 3). The scheme of dialysis for each patient with DGF is depicted in eFigure 1 in Supplement 2. In the Kaplan-Meier analysis, the risk of DGF was significantly lower in the dexmedetomidine group (HR, 0.48; 95% CI, 0.23-0.99; *P* = .04) (eFigure 2 in Supplement 2).

**Table 3.** Study Outcomes

Outcome	Patients, No. (%)		OR, Difference, or HR (95% CI)	*P* value
	Dexmedetomidine group (n = 56)	Normal saline group (n = 55)		
**Primary^a^**				
DGF incidence in first posttransplant week	10 (17.9)	19 (34.5)	OR, 0.41 (0.17 to 0.98)	.04
**Secondary^b^**				
In-hospital				
Repeated dialysis in first posttransplant week	7 (12.5)	17 (30.9)	OR, 0.32 (0.13 to 0.88)	.02
Acute rejection	5 (8.9)	7 (12.7)	OR, 0.67 (0.22 to 2.14)	.52
30-d Posttransplant				
Serum creatinine, mean (SD), mg/dL	1.42 (0.92)	1.57 (1.39)	Difference, −0.15 (−0.60 to 0.29)	.50
Serum cystatin C, median (IQR), mg/L	1.74 (1.26 to 2.28)	1.75 (1.35 to 2.29)	Difference, −0.01 (−0.31 to 0.17)	.65
eGFR, mean (SD), mL/min/1.73 m_2_	65.1 (24.1)	63.3 (27.4)	Difference, 1.82 (−7.89 to 11.52)	.71
Need for dialysis	1 (1.8)	3 (5.5)	OR, 0.32 (0.03 to 3.13)	.36
Patient survival	56 (100)	55 (100)	NA	>.99
**Post hoc^c^**				
In-hospital				
CRR2, median (IQR), %	36.5 (1.4 to 53.5)	23.6 (−0.1 to 51.2)	Difference, 12.9 (−7.8 to 14.7)	.65
CRR2 <30%	26 (46.4)	31 (56.4)	OR, 0.67 (0.32 to 1.38)	.29
No. of dialysis in first posttransplant week, median (IQR)	2 (1 to 5) [n = 10]	3 (2 to 5) [n = 19]	Difference, −1 (−2 to 1)	.49
Timing of acute rejection diagnosis, mean (SD), POD	15.0 (5.2) [n = 5]	12.4 (2.8) [n = 7]	Difference, 2.6 (−3.8 to 8.9)	.35
Acute rejection in first posttransplant week	0	0	NA	>.99
Pneumonia	2 (3.6)	5 (9.1)	OR, 0.37 (0.07 to 2.00)	.27
DVT	0	0	NA	>.99
Subgroups, No. (%) [total No.]				
DGF incidence in donor criteria subgroups				
Expanded-criteria donor	1 (25) [4]	2 (50) [4]	OR, 0.33 (0.02 to 5.17)	>.99
Standard-criteria donor	9 (17.3) [52]	17 (33.3) [51]	OR, 0.42 (0.18 to 1.08)	.06
DGF in KDRI subgroups, KDRI quintiles				
1-3	5 (19.2) [26]	8 (34.8) [23]	OR, 0.45 (0.13 to 1.60)	.33
4-5	5 (16.7) [30]	11 (34.4) [32]	OR, 0.38 (0.13 to 1.29)	.15
1-y Posttransplant outcomes				
Serum creatinine, mean (SD), mg/dL	1.18 (0.35)	1.57 (1.83) [n = 54]	Difference, −0.39 (−0.88 to 0.11)	.12
Serum cystatin C, median (IQR), mg/L	1.25 (1.08 to 1.67)	1.35 (1.08 to 1.54) [n = 54]	Difference, −0.10 (−0.18 to 0.13)	.80
eGFR, mean (SD), mL/min	70.6 (20.2)	66.8 (24.3) [n = 54]	Difference, 3.8 (−4.6 to 12.3)	.37
Acute rejection^d^	6 (10.7)	8 (14.5)	HR, 0.71 (0.25 to 2.04)	.53
Allograft failure^d^	0	3 (5.5)	HR, 0.13 (0.01 to 1.26)	.08
Patient survival^d^	56 (100)	55 (100)	NA	>.99

Abbreviations: CRR2, creatinine reduction ratio on posttransplant day 2; DGF, delayed graft function; DVT, deep vein thrombosis; eGFR, estimated glomerular filtration rate; HR, hazard ratio; KDRI, Kidney Donor Risk Index; NA, not applicable; OR, odds ratio; POD, postoperative day.

SI conversion factor: To convert serum creatinine levels to micromole per liter, multiply by 88.4.

^a^ For the primary outcome, statistical significance was *P* < .05.

^b^ For the secondary outcomes, statistical significance was *P* < .007 after multiple testing correction.

^c^ No multiple testing correction was planned a priori; thus, these data should be interpreted as exploratory.

^d^ *P* value was calculated by Kaplan-Meier curves with log-rank tests.

### Secondary Outcomes

Dexmedetomidine reduced the need for repeated dialysis during the first posttransplant week (7 of 56 [12.5%] vs 17 of 55 [30.9%]; OR, 0.32; 95% CI, 0.13-0.88; *P* = .02) (Table 3), without significant between-group difference after multiple testing corrections. In-hospital biopsy-proven acute rejection occurred in 5 patients (8.9%) in the dexmedetomidine group and 7 patients (12.7%) in the normal saline group. One patient (1.8%) in the dexmedetomidine group and 3 patients (5.5%) in the normal saline group needed dialysis on posttransplant day 30.

### Post Hoc Analyses

The median (IQR) creatinine reduction ratio on posttransplant day 2 was 36.5% (1.4%-53.5%) in the dexmedetomidine group and 23.6% (−0.1% to 51.2%) in the normal saline group (difference, 12.9%; 95% CI, −7.8% to 14.7%) (Table 3). The proportion of patients with a creatinine reduction ratio on posttransplant day 2 less than 30% was 46.4% in the dexmedetomidine group vs 56.4% in the normal saline group. Acute rejection was diagnosed on mean (SD) POD 15.0 (5.2) in the dexmedetomidine group and 12.4 (2.8) in the normal saline group. Two patients (3.6%) in the dexmedetomidine group and 5 (9.1%) in the normal saline group developed pneumonia. No patient had deep vein thrombosis. The effect of dexmedetomidine on DGF was similar in donor criteria subgroups (expanded-criteria donor vs standard-criteria donor) and KDRI subgroups (KDRI quintiles 1-3 vs KDRI quintiles 4-5).

At 1 year postoperatively, the mean (SD) serum creatinine level was 1.18 (0.35) mg/dL in the dexmedetomidine group and 1.57 (1.83) mg/dL in the normal saline group (difference, −0.39 mg/dL; 95% CI, −0.88 to 0.11 mg/dL) (Table 3); to convert serum creatinine levels to micromole per liter, multiply by 88.4. Of the 4 patients with a 30-day posttransplant need for dialysis, 3 patients (5.5%) in the normal saline group were still on regular dialysis at 1 year postoperatively, and 1 patient (1.8%) in the dexmedetomidine group had the last dialysis on posttransplant day 43 and did not require dialysis after that. The Kaplan-Meier analysis of 1-year allograft failure showed an HR of 0.13 (95% CI, 0.01-1.26) for the dexmedetomidine group vs the normal saline group.

## Discussion

To our knowledge, this trial was the first to show that 24-hour perioperative dexmedetomidine administration reduced the incidence of DGF after DCD kidney transplant. Decreased need for repeated dialysis further suggested the favorable effects of dexmedetomidine on kidney allograft during the first posttransplant week, a higher creatinine clearance rate on PODs 1 and 2, and increased urine output on PODs 2 and 7 and at hospital discharge. The dexmedetomidine infusion neither led to concerning adverse events nor adversely affected postoperative recovery.

Delayed graft function is associated with an increased risk of acute rejection, inferior graft function, prolonged hospital stay, and reduced long-term graft survival and patient survival.^6,8,35^ Interventions, including eculizumab, dopamine, epoetin alfa, and hypothermic machine perfusion, had minimal or no effect on reducing DGF after kidney transplant.^36,37,38,39^ A retrospective cohort study found an association between dexmedetomidine and decreased incidence of DGF, overall complications, infection, acute rejection in the early posttransplant phase, and length of hospital stay.^13^ That study had several limitations, however, including its cohort design, the heterogenous donor sources, the mixture of kidney-only and combined kidney-pancreas transplants, lack of multiple testing corrections, and potentially inadequate confounder control.

In contrast, the present trial was based on a randomized design and showed the DGF reduction effect exerted by dexmedetomidine in DCD kidney transplant. The finding was corroborated by previous studies reporting that dexmedetomidine decreased acute kidney injury in cardiac surgery.^25,40^ Dexmedetomidine has a favorable safety profile and is currently used in perioperative and critical care. A recent study found an association between dexmedetomidine and improved 5-year survival after cardiac surgery.^34^ The infusion rate and duration of dexmedetomidine in this trial were in concordance with those in current practice. Dexmedetomidine infusion (0.4 μg/kg/h intraoperatively and 0.1 μg/kg/h postoperatively) has been used in the perioperative setting.^25,26,40^ The low infusion rate may enhance postoperative recovery via anxiolysis, analgesia, sleeping promotion, and delirium reduction.^26^ The pilot study and this trial found neither concerning adverse effects nor delayed postoperative recovery associated with dexmedetomidine. The 1-year postoperative outcomes suggested that the dexmedetomidine treatment may improve longer-term kidney allograft function. The dexmedetomidine group had lower serum creatinine and cystatin C levels, higher eGFR, and a lower allograft failure rate up to 1-year postoperatively, although these between-group differences were not statistically significant. The present trial was likely underpowered for these long-term outcomes. The overall risk-benefit profile supports the use of dexmedetomidine in kidney transplants.

Several potential mechanisms may underlie the favorable effects of dexmedetomidine on kidney transplants. As an α_2_-adrenoreceptor agonist, dexmedetomidine engages in the α_2_-adrenoreceptors that are widely populated in kidney tubules and peritubular vascular structures. The activation of the α_2_-adrenoreceptor pathway decreases sympathoadrenal hyperactivity. It induces vasodilatation via endothelial nitric oxide regulation, leading to enhanced glomerular filtration and increased urine output.^41,42^ Preclinical studies reported that dexmedetomidine attenuated inflammation, reduced kidney endothelial chemokines, inhibited reperfusion-induced cell death signaling, and enhanced cell survival signaling.^10,11,43,44^ A meta-analysis found that dexmedetomidine as an adjuvant during anesthesia attenuated surgical stress and inflammation,^45^ which may enhance postoperative recovery and improve overall outcomes.

### Limitations

This trial has several limitations. First, although the sample size agreed with the result of the power analysis, including more patients would have enhanced the power. Second, the long-term outcomes were based on post hoc analysis. Third, although dexmedetomidine may reduce the length of hospital stay after kidney transplant,^13^ we did not observe such an effect. The median length of hospital stay of 25 days in this trial was in line with the data originated in China^46,47^ but appeared much longer than that in the US and European countries.^48,49,50^ This difference may be attributed to the different health care systems in various countries. Fourth, as a single-center trial based on controlled donors after cardiac death and characterized by a relatively short warm ischemic time, the generalizability of its findings should be tested in future studies.

## Conclusions

In this randomized clinical trial, the 24-hour perioperative dexmedetomidine infusion reduced the incidence of DGF without incurring adverse effects after DCD kidney transplant. The findings of this trial support the use of dexmedetomidine in kidney transplants. Further trials are needed to determine the effects of dexmedetomidine on long-term outcomes and on different kidney transplant scenarios.

## Notes

Supplement 1.
Trial Protocol

Supplement 2. **eMethods.** Evolutionary Phases of Organ Transplantation in China, Organ Donation and Procurement, Risk Assessment of Donor’s Kidneys, Anesthetic Care, Perioperative Practice of Dialysis Treatment, Measurements of Creatinine Clearance Rate and Urine Output, and Immunosuppressive Medications **eTable. **Results of Postoperative Graft Function-Related Parameters **eFigure 1.** Scheme of Dialysis for Each Patient With Delayed Graft Function **eFigure 2.** Kaplan-Meier Plot for Delayed Graft Function During 0-7 Days After Kidney Transplantation

Supplement 3.
Data Sharing Statement

## References

[1] United States Renal Data System. 2018 USRDS annual data report: epidemiology of kidney disease in the United States. National Institutes of Health, National Institute of Diabetes and Digestive and Kidney Diseases; 2018. Accessed February 9, 2021. https://nccd.cdc.gov/ckd/detail.aspx?Qnum=Q67

[2] Husain SA, Chiles MC, Lee S, . Characteristics and performance of unilateral kidney transplants from deceased donors. Clin J Am Soc Nephrol. 2018;13(1):118-127. doi:10.2215/CJN.06550617 29217537 PMC5753314

[3] Axelrod DA, Schnitzler MA, Xiao H, . An economic assessment of contemporary kidney transplant practice. Am J Transplant. 2018;18(5):1168-1176. doi:10.1111/ajt.14702 29451350

[4] Kidney Disease: Improving Global Outcomes (KDIGO) Transplant Work Group. KDIGO clinical practice guideline for the care of kidney transplant recipients. Am J Transplant. 2009;9(suppl 3):S1-S155. doi:10.1111/j.1600-6143.2009.02834.x 19845597

[5] Lim MA, Bloom RD. Medical therapies to reduce delayed graft function and improve long-term graft survival: are we making progress? Clin J Am Soc Nephrol. 2020;15(1):13-15. doi:10.2215/CJN.13961119 31911413 PMC6946075

[6] Wu WK, Famure O, Li Y, Kim SJ. Delayed graft function and the risk of acute rejection in the modern era of kidney transplantation. Kidney Int. 2015;88(4):851-858. doi:10.1038/ki.2015.190 26108067

[7] Ponticelli C. Ischaemia-reperfusion injury: a major protagonist in kidney transplantation. Nephrol Dial Transplant. 2014;29(6):1134-1140. doi:10.1093/ndt/gft488 24335382

[8] Chen R, Wang H, Song L, . Predictors and one-year outcomes of patients with delayed graft function after deceased donor kidney transplantation. BMC Nephrol. 2020;21(1):526. doi:10.1186/s12882-020-02181-1 33276737 PMC7716446

[9] Gerlach AT, Murphy CV, Dasta JF. An updated focused review of dexmedetomidine in adults. Ann Pharmacother. 2009;43(12):2064-2074. doi:10.1345/aph.1M310 19934395

[10] Ma J, Chen Q, Li J, . Dexmedetomidine-mediated prevention of renal ischemia-reperfusion injury depends in part on cholinergic anti-inflammatory mechanisms. Anesth Analg. 2020;130(4):1054-1062. doi:10.1213/ANE.0000000000003820 30346356

[11] Kim SH, Jun JH, Oh JE, Shin EJ, Oh YJ, Choi YS. Renoprotective effects of dexmedetomidine against ischemia-reperfusion injury in streptozotocin-induced diabetic rats. PLoS One. 2018;13(8):e0198307. doi:10.1371/journal.pone.0198307 30114208 PMC6095484

[12] Peng K, Li D, Applegate RL II, Lubarsky DA, Ji FH, Liu H. Effect of dexmedetomidine on cardiac surgery-associated acute kidney injury: a meta-analysis with trial sequential analysis of randomized controlled trials. J Cardiothorac Vasc Anesth. 2020;34(3):603-613. doi:10.1053/j.jvca.2019.09.011 31587928

[13] Chen J, Perez R, de Mattos AM, . Perioperative dexmedetomidine improves outcomes of kidney transplant. Clin Transl Sci. 2020;13(6):1279-1287. doi:10.1111/cts.12826 32506659 PMC7719359

[14] Chinese Society of Organ Transplantation, Chinese Medical Association. National guidelines for donation after cardiac death in China. Hepatobiliary Pancreat Dis Int. 2013;12(3):234-238. doi:10.1016/S1499-3872(13)60038-7 23742766

[15] Sánchez-Fructuoso AI, Prats D, Torrente J, . Renal transplantation from non-heart beating donors: a promising alternative to enlarge the donor pool. J Am Soc Nephrol. 2000;11(2):350-358. doi:10.1681/ASN.V112350 10665943

[16] Huang J, Millis JM, Mao Y, Millis MA, Sang X, Zhong S. A pilot programme of organ donation after cardiac death in China. Lancet. 2012;379(9818):862-865. doi:10.1016/S0140-6736(11)61086-6 22078722

[17] Lewis J, Peltier J, Nelson H, . Development of the University of Wisconsin donation after cardiac death evaluation tool. Prog Transplant. 2003;13(4):265-273. doi:10.1177/152692480301300405 14765718

[18] Suntharalingam C, Sharples L, Dudley C, Bradley JA, Watson CJ. Time to cardiac death after withdrawal of life-sustaining treatment in potential organ donors. Am J Transplant. 2009;9(9):2157-2165. doi:10.1111/j.1600-6143.2009.02758.x 19681825

[19] Debout A, Foucher Y, Trébern-Launay K, . Each additional hour of cold ischemia time significantly increases the risk of graft failure and mortality following renal transplantation. Kidney Int. 2015;87(2):343-349. doi:10.1038/ki.2014.304 25229341

[20] Ethier I, Cho Y, Hawley C, . Multicenter registry analysis comparing survival on home hemodialysis and kidney transplant recipients in Australia and New Zealand. Nephrol Dial Transplant. 2021;36(10):1937-1946. doi:10.1093/ndt/gfaa159 32879952

[21] Clayton PA, Dansie K, Sypek MP, . External validation of the US and UK kidney donor risk indices for deceased donor kidney transplant survival in the Australian and New Zealand population. Nephrol Dial Transplant. 2019;34(12):2127-2131. doi:10.1093/ndt/gfz090 31157885

[22] Rao PS, Schaubel DE, Guidinger MK, . A comprehensive risk quantification score for deceased donor kidneys: the kidney donor risk index. Transplantation. 2009;88(2):231-236. doi:10.1097/TP.0b013e3181ac620b 19623019

[23] Xue W, Wang C, Chen J, . A prediction model of delayed graft function in deceased donor for renal transplant: a multi-center study from China. Ren Fail. 2021;43(1):520-529. doi:10.1080/0886022X.2021.1895838 33719820 PMC7971200

[24] Kasiske BL, Zeier MG, Chapman JR, ; Kidney Disease: Improving Global Outcomes. KDIGO clinical practice guideline for the care of kidney transplant recipients: a summary. Kidney Int. 2010;77(4):299-311. doi:10.1038/ki.2009.377 19847156

[25] Cho JS, Shim JK, Soh S, Kim MK, Kwak YL. Perioperative dexmedetomidine reduces the incidence and severity of acute kidney injury following valvular heart surgery. Kidney Int. 2016;89(3):693-700. doi:10.1038/ki.2015.306 26444030

[26] Su X, Meng ZT, Wu XH, . Dexmedetomidine for prevention of delirium in elderly patients after non-cardiac surgery: a randomised, double-blind, placebo-controlled trial. Lancet. 2016;388(10054):1893-1902. doi:10.1016/S0140-6736(16)30580-3 27542303

[27] Schnuelle P, Gottmann U, Hoeger S, . Effects of donor pretreatment with dopamine on graft function after kidney transplantation: a randomized controlled trial. JAMA. 2009;302(10):1067-1075. doi:10.1001/jama.2009.1310 19738091

[28] Levey AS, Stevens LA, Schmid CH, ; CKD-EPI (Chronic Kidney Disease Epidemiology Collaboration). A new equation to estimate glomerular filtration rate. Ann Intern Med. 2009;150(9):604-612. doi:10.7326/0003-4819-150-9-200905050-00006 19414839 PMC2763564

[29] Rodrigo E, Ruiz JC, Piñera C, . Creatinine reduction ratio on post-transplant day two as criterion in defining delayed graft function. Am J Transplant. 2004;4(7):1163-1169. doi:10.1111/j.1600-6143.2004.00488.x 15196076

[30] Yarlagadda SG, Coca SG, Garg AX, . Marked variation in the definition and diagnosis of delayed graft function: a systematic review. Nephrol Dial Transplant. 2008;23(9):2995-3003. doi:10.1093/ndt/gfn158 18408075 PMC2727302

[31] Zens TJ, Danobeitia JS, Leverson G, . The impact of kidney donor profile index on delayed graft function and transplant outcomes: a single-center analysis. Clin Transplant. 2018;32(3):e13190. doi:10.1111/ctr.13190 29314286 PMC6455919

[32] Iyasere OU, Brown EA, Johansson L, . Quality of life and physical function in older patients on dialysis: a comparison of assisted peritoneal dialysis with hemodialysis. Clin J Am Soc Nephrol. 2016;11(3):423-430. doi:10.2215/CJN.01050115 26712808 PMC4785682

[33] Li F, Li D, Yu J, . Barthel index as a predictor of mortality in patients with acute coronary syndrome: better activities of daily living, better prognosis. Clin Interv Aging. 2020;15:1951-1961. doi:10.2147/CIA.S270101 33116449 PMC7568594

[34] Peng K, Shen YP, Ying YY, . Perioperative dexmedetomidine and 5-year survival in patients undergoing cardiac surgery. Br J Anaesth. 2021;127(2):215-223. doi:10.1016/j.bja.2021.03.040 34082896

[35] Lim WH, Johnson DW, Teixeira-Pinto A, Wong G. Association between duration of delayed graft function, acute rejection, and allograft outcome after deceased donor kidney transplantation. Transplantation. 2019;103(2):412-419. doi:10.1097/TP.0000000000002275 29762458

[36] Schröppel B, Akalin E, Baweja M, . Peritransplant eculizumab does not prevent delayed graft function in deceased donor kidney transplant recipients: results of two randomized controlled pilot trials. Am J Transplant. 2020;20(2):564-572. doi:10.1111/ajt.15580 31452319

[37] Tedesco-Silva H, Mello Offerni JC, Ayres Carneiro V, . Randomized trial of machine perfusion versus cold storage in recipients of deceased donor kidney transplants with high incidence of delayed graft function. Transplant Direct. 2017;3(5):e155. doi:10.1097/TXD.0000000000000672 28573190 PMC5441986

[38] Schnuelle P, Schmitt WH, Weiss C, . Effects of dopamine donor pretreatment on graft survival after kidney transplantation: a randomized trial. Clin J Am Soc Nephrol. 2017;12(3):493-501. doi:10.2215/CJN.07600716 28213388 PMC5338714

[39] Sureshkumar KK, Hussain SM, Ko TY, Thai NL, Marcus RJ. Effect of high-dose erythropoietin on graft function after kidney transplantation: a randomized, double-blind clinical trial. Clin J Am Soc Nephrol. 2012;7(9):1498-1506. doi:10.2215/CJN.01360212 22745272 PMC3430945

[40] Soh S, Shim JK, Song JW, Bae JC, Kwak YL. Effect of dexmedetomidine on acute kidney injury after aortic surgery: a single-centre, placebo-controlled, randomised controlled trial. Br J Anaesth. 2020;124(4):386-394. doi:10.1016/j.bja.2019.12.036 32007239

[41] Nong L, Ma J, Zhang G, . Dexmedetomidine inhibits vasoconstriction via activation of endothelial nitric oxide synthase. Korean J Physiol Pharmacol. 2016;20(5):441-447. doi:10.4196/kjpp.2016.20.5.441 27610030 PMC5014990

[42] Ebert TJ, Hall JE, Barney JA, Uhrich TD, Colinco MD. The effects of increasing plasma concentrations of dexmedetomidine in humans. Anesthesiology. 2000;93(2):382-394. doi:10.1097/00000542-200008000-00016 10910487

[43] Lempiäinen J, Finckenberg P, Mervaala EE, . Dexmedetomidine preconditioning ameliorates kidney ischemia-reperfusion injury. Pharmacol Res Perspect. 2014;2(3):e00045. doi:10.1002/prp2.45 25505591 PMC4186414

[44] Ueki M, Kawasaki T, Habe K, Hamada K, Kawasaki C, Sata T. The effects of dexmedetomidine on inflammatory mediators after cardiopulmonary bypass. Anaesthesia. 2014;69(7):693-700. doi:10.1111/anae.12636 24773263

[45] Wang K, Wu M, Xu J, . Effects of dexmedetomidine on perioperative stress, inflammation, and immune function: systematic review and meta-analysis. Br J Anaesth. 2019;123(6):777-794. doi:10.1016/j.bja.2019.07.027 31668347

[46] Chu A, Zhang T, Fang Y, Yuan L, Guan X, Zhang H. Unplanned hospital readmissions after kidney transplantation among patients in Hefei, China: incidence, causes and risk factors. Int J Nurs Sci. 2020;7(3):291-296. doi:10.1016/j.ijnss.2020.05.002 32817851 PMC7424151

[47] Na N, Li K, Huang Z, . Posttransplant outcomes of kidneys donated after brain death followed by circulatory death: a cohort study of 128 Chinese patients. Transplant Direct. 2017;3(8):e189. doi:10.1097/TXD.0000000000000704 28795141 PMC5540627

[48] Raza MH, Jackson WE, Dell A, . Health-related quality of life after anonymous nondirected living liver donation: a multicenter collaboration. Am J Transplant. 2021;21(3):1056-1067. doi:10.1111/ajt.16229 32741102 PMC8351218

[49] Sussell J, Silverstein AR, Goutam P, . The economic burden of kidney graft failure in the United States. Am J Transplant. 2020;20(5):1323-1333. doi:10.1111/ajt.15750 32020739

[50] Veighey KV, Nicholas JM, Clayton T, . Early remote ischaemic preconditioning leads to sustained improvement in allograft function after live donor kidney transplantation: long-term outcomes in the Renal Protection Against Ischaemia-Reperfusion in Transplantation (REPAIR) randomised trial. Br J Anaesth. 2019;123(5):584-591. doi:10.1016/j.bja.2019.07.019 31521337

