# Supplementary material for: Effect of Perioperative Dexmedetomidine on Delayed Graft Function Following a Donation-After-Cardiac-Death Kidney Transplant: A Randomized Clinical Trial
Source: JAMA Netw Open. 2022 Jun 3;5(6):e2215217. doi: 10.1001/jamanetworkopen.2022.15217 (PMC9166619; doi:10.1001/jamanetworkopen.2022.15217)
Supplement: Supplement 1. — Trial Protocol [file jamanetwopen-e2215217-s001.pdf]

Effect of perioperative dexmedetomidine on delayed graft function after donation after cardiac death kidney transplantation

A randomized double-blind placebo-controlled trial

**Title**	Effect of perioperative dexmedetomidine on delayed graft function after donation after cardiac death kidney transplantation: a randomized double-blind placebo-controlled trial
**Institution**	First Affiliated Hospital of Soochow University
**Principal Investigator**	Fu-hai Ji, MD, PhD
**Address of Principle Investigator**	Department of Anesthesiology<br>First Affiliated Hospital of Soochow University<br>899 Pinghai Rd, Suzhou, Jiangsu, 215006, China<br>Tel: +86-512-6797-2352; Email: jifuhaisuda@163.com<br>ORCID: 0000-0001-6649-665X
**Concept and Design**	Fu-hai Ji, Ke Peng, Lingzhong Meng
**Study Contact**	Ke Peng<br>Address: 899 Pinghai Rd, Suzhou, Jiangsu, 215006, China<br>Tel: +86-15962155989; Email: pengke0422@163.com
**Version**	1.2
**Date**	2019-06-20

Table of Contents

1. Synopsis 4
2. Flow Chart 6
3. Glossary 7
4. Background and Rationale 9
   4.1 End stage renal disease and kidney transplantation 9
   4.2 Delayed graft function (DGF) 9
   4.3 Dexmedetomidine in kidney transplantation 10
   4.4 Our pilot data 11
   4.5 The aim of this study 11
5. Study Design 12
   5.1 Description of this study 12
   5.2 Duration of this study 12
6. Study Outcomes and Perioperative Data 14
   6.1 Primary outcome 14
   6.2 Secondary outcomes 14
   6.3 Perioperative data 14
7. Selection of Patients 15
   7.1 Inclusion criteria 15
   7.2 Exclusion criteria 15
8. Study Treatment 16
   8.1 Randomization and blinding 16
   8.2 Anesthesia management 16
   8.3 Study drug interventions 17
   8.4 Accountability and compliance 18
9. Safety Concerns 19
   9.1 Safety profile of dexmedetomidine 19
   9.2 Hemodynamic responses and monitoring 19
   9.3 Discontinuation of intervention and unmasking 19
10. Statistical Plan 21
   10.1 Sample size estimation 21

10.2 Statistical analysis 21
10.3 Statisticians 22
11. Ethical Considerations 23
11.1 Ethical approval and clinical trial registration 23
11.2 Informed consent 23
11.3 Responsibilities 23
12. Data Management and Clinical Trial Report 24
12.1 Source documents 24
12.2 Case Report Forms (CRFs) 24
12.3 Data protection 24
12.4 Clinical trial report 24
13. References 25

1. Synopsis

**Study Title**	Effect of perioperative dexmedetomidine on delayed graft function after donation after cardiac death kidney transplantation: a randomized double-blind placebo-controlled trial
**Study Design**	Researcher-initiated, prospective, randomized (1:1), double-blind, placebo-controlled trial
**Principal Investigator**	Fu-hai Ji, MD, PhD
**Trial Site**	Department of Anesthesiology<br>First Affiliated Hospital of Soochow University<br>899 Pinghai Rd, Suzhou, Jiangsu, 215006, China
**Selection Criteria**	**Inclusion Criteria:**<br>1. Age ≥ 18 years;<br>2. Diagnosed with end-stage renal disease;<br>3. Scheduled for donation after cardiac death (DCD) kidney transplant under general anesthesia.<br>**Exclusion Criteria:**<br>1. Sick sinus syndrome;<br>2. Atrioventricular block;<br>3. A left ventricular ejection fraction < 30%;<br>4. Multi-organ transplantation.
**Study Outcomes**	**Primary:**<br>The incidence of DGF, defined as the need for dialysis during the first week after kidney transplant.
	**Secondary:**<br>— Repeated dialysis during the first post-transplant week;<br>— In-hospital acute rejection;<br>— 30-day serum creatinine;<br>— 30-day serum cystatin C;<br>— 30-day estimated glomerular filtration rate (eGFR);<br>— 30-day allograft survival;<br>— 30-day patient survival.
**Expected Number of Patients**	114 donation after cardiac death kidney transplant recipients, with 57 in each arm (dexmedetomidine vs. saline)
**Study Drug and Interventions**	Patients will be randomized into a dexmedetomidine group to receive intravenous dexmedetomidine infusion for a total of 24 hours (0.4 μg/kg/h, starting immediately after anesthesia

	induction and continuing during procedure, followed by 0.1 μg/kg/h after surgery in a transplant care unit) or a normal saline group to receive saline, with the same administration as for dexmedetomidine.
**Safety Concerns**	No additional risk associated with dexmedetomidine use for the patients is expected.<br>— Hypotension and bradycardia are potential hemodynamic side-effects of dexmedetomidine, but most studies found these effects to be self-limiting and clinically benign.<br>— To avoid potential bradycardia, no bolus dose of dexmedetomidine is given.
**Statistical Plan**	Sample size is calculated based on our preliminary data and previous studies. We hypothesize that the incidence of DGF would be reduced by 50% after dexmedetomidine treatment. To detect such a difference with a power of 80% at an alpha level of 0.05, the sample size is estimated to be 108 patients, with an allocation ratio of 1:1. Considering that 5% of the subjects might be lost to follow-up or withdraw their informed consent, we decide to enroll a total of 114 patients (57 patients in each group).<br>All data will be analyzed according to the modified intention-to-treat principle, including any randomized patient with their result of the primary outcome available. Analyses will be performed with the use of SPSS (version 19.0, IBM SPSS, Chicago, IL) and GraphPad Prism (version 7.00; GraphPad, San Diego, CA), according to a prespecified analysis plan. Neither an interim analysis nor an imputation of missing data is planned.<br>The DGF incidence will be analyzed using the Chi-square test, and the Kaplan–Meier curve will be also constructed. A two-sided $P < 0.05$ indicates a statistically significant difference. The secondary outcomes will be analyzed using the independent t test, Mann–Whitney rank-sum test, Chi-square test or Fisher exact test, as appropriate. The therapeutic effect will be assessed using odds ratio (or difference) and 95% confidence intervals. Multiple testing for the secondary outcomes will be corrected using the Bonferroni method, with P value < 0.007 indicating a statistically significant difference.
**Study Period**	30 days after the transplant surgery

2. Flow Chart

	Screening	Study Phase			
		Treatment	Post treatment follow-up		
	Before surgery	In OR	Day 1 to 7 in transplant unit	Hospital discharge	30 days postoperatively
Evaluation					
Inclusion/Exclusion Criteria	×				
Medical/Surgical History	×				
Informed Consent	×				
Prior Medication History	×				
Patient Demographics	×				
Clinical Examination	×				
Vital Signs	×				
Lab Testing	×				
Randomization		×			
Treatment					
Intervention		×			
Compliance		×			
Outcome evaluation					
Primary			×		
Secondary			×	×	×
Lab Testing			×	×	×
Safety					
AE/SAE (if any)		×	×	×	×

AE, adverse event; SAE, serious adverse event.

3. Glossary

- **Good Clinical Practice (GCP)**

An international standard of ethics and scientific quality to design, conduct, register and confront studies on human subjects. Adherence to these standards not only guarantees the safety, the well-being, and the rights of the participates, in accordance with the Helsinki Declaration (1947) principles, but also the reliability of the study data.

- **Ethic Committee (EC)**

An independent organization, composed by health care and non-heath care staff that is responsible for the safeguard of the safety, the well-being, and the rights of study subjects and that must also publicly guarantee this safeguard by, for example, expressing an opinion on the experimental protocol, on the investigators, on the adequacy of the structures, methods and documents used to inform the subjects and obtain informed consent.

- **Clinical Research Office (CRO)**

The Clinical Research Office takes care of the preparation and revision of all documents involved in research protocols (whether sponsored, internal, or part of a study group), including amendments and modifications, that are then analyzed by the EC at First Affiliated Hospital of Soochow University to be approved and then authorized. The CRO works as a scientific and technical secretariat of the EC.

- **No Profit Study**

No profit study does not aim at the industrial development of a drug or, in any case, not for profit, but aims at improving clinical practice. This objective must be guaranteed by the protocol relevance, by the peculiarity of the disease, and by the type of treatment.

- **Investigator**

A physician qualified in clinical research is responsible for this clinical trial in a university hospital. If the trial is followed by a group of investigators in the same hospital, the investigator who is responsible is called “Principal Investigator” (PI).

- **Case Report Form (CRF)**

The Case Report Form (CRF) is a printed or digital document designed to register all the information required by the study protocol that must be reported regarding each study subject. It is usually not an original document.

- **Adverse event (AE)**

Any negative clinical event that involves a patient undergoing a clinical trial who has received a study drug, even though the event does not necessarily have a causative correlation with the treatment.

- **Serious Adverse event (SAE)**

Any adverse event or drug reaction that, regardless of the dose, causes the patient's death or threatens the subject's life, requires hospitalization, or prolongs hospitalization, or causes prolonged or severe invalidity, or involved a congenital anomaly or a birth problem.

4. Background and Rationale

4.1 End stage renal disease and kidney transplantation

As of December 31, 2016, 726,331 patients were treated for end stage renal disease (ESRD) in the United States, giving a point prevalence of 2,206 per million population; prevalence of dialysis treatment was 1,553 per million, whereas prevalence of functioning kidney transplant was 653 per million. The prevalence of ESRD more than doubled between 1990 and 2016.[1] Kidney transplantation is an established effective treatment for end-stage renal disease,[2,3] with the number of patients receiving allograft kidney transplantation in the United States increasing from 10,011 in 1991 to 19,355 in 2016.[1] Kidney transplantation leads to improved survival, better quality of life, and reduced healthcare cost when compared with regular dialysis in patients with ESRD.[2-4] China has established a legal and procedural framework for an organ donation system based on voluntary donation after cardiac death that adheres to both China's social and cultural principles and international transplantation standards.[5,6]

4.2 Delayed graft function (DGF)

Nonetheless, various complications still complicate post-transplant course in patients undergoing kidney allotransplantation, such as delayed graft function (DGF) which is defined as the need for dialysis during the first week after kidney transplantation.[7] DGF is a major complication after kidney transplantation, and has an incidence of 4%–10% in living and up to 50% in deceased donor kidney transplant recipients. [7-9] Most importantly, DGF is associated with increased risk of acute rejection and reduced long-term graft survival.[9] Therefore, DGF following kidney transplantation remains a major challenge. Given its clinical significance, DGF is commonly used as the endpoint in kidney transplantation research.

The occurrence of DGF can be attributed to ischemia-reperfusion injury, oxidative stress, excessive inflammatory reaction, and innate immune response.[10,11] To date, however, there is no Food and Drug Administration (FDA)-approved therapy for DGF, and most published studies showed minimal or no effect of the interventions (e.g., eculizumab, dopamine, epoetin-α, recombinant P-selectin glycoprotein ligand IgG fusion protein, hypothermic machine perfusion) on DGF or graft survival after kidney transplantation.[12-15] A recent promising result is that treatment of with C1 esterase

inhibitor was associated with a reduced rate of DGF after deceased donor kidney transplantation.[16] However, the sample size is small (35 patients each in the C1 esterase inhibitor and placebo groups); therefore, more evidence is certainly needed in terms of the efficacy and safety of this treatment for preventing DGF.

4.3 Dexmedetomidine in kidney transplantation

Dexmedetomidine is a selective and specific presynaptic and postsynaptic α2-adrenoreceptor agonist with an α2: α1 receptor affinity of 1620:1.[17] Dexmedetomidine possibly mediates both sympatholytic and vasoconstrictive hemodynamic effects.[18] It exerts sedating and anxiolytic action through presynaptic α2-adrenergic receptor stimulation of the locus coeruleus in the brainstem. The locus coeruleus also is the origin of the descending adrenergic component of the spinal cord, known to be the key pathway intervening in the regulation of nociceptive neurotransmission responsible for the analgesic effect of dexmedetomidine.[19] Studies evaluating the hemodynamic stabilizing and sympatholytic effects have shown that α-2 agonists can potentially reduce postoperative cardiovascular complications[20,21]. Dexmedetomidine was first approved by the FDA in 1999 as a sedative, and now dexmedetomidine has been a focus of extensive research because of its many beneficial effects.

Dexmedetomidine has been shown to attenuate ischemia-reperfusion injury in animal studies.[22-24] It also attenuates inflammatory responses and surgical stress, and protects the immune function.[25-27] Recent studies have suggested that, for patients who underwent cardiac surgery, dexmedetomidine reduced the incidence and severity of acute kidney injury (AKI), without significant hemodynamic adverse effects.[28] Multiple studies have reported that dexmedetomidine has protective effects on specific organs, including the heart, brain, kidney, and lungs.[29,30] Based on these, it would be reasonable to postulate that perioperative dexmedetomidine use might confer benefits for patients undergoing kidney transplantation, given the well-proven benefits of sympatholytic, anti-inflammatory, and anti-delirium effects associated with dexmedetomidine. However, there is no study investigating dexmedetomidine use on graft outcomes after kidney transplantation. We generate the hypothesis that perioperative use of dexmedetomidine will improve kidney graft function in patients undergoing kidney transplantation.

4.4 Our pilot data

In our institution, the pilot data shows that 9 out of 20 (45%) DCD kidney transplant recipients who did not receive dexmedetomidine experienced DGF during the first week after kidney transplantation. However, the therapeutic effect of dexmedetomidine on DGF after DCD kidney transplantation is still unknown.

4.5 The aim of this study

To date, there is no randomized study to investigate whether dexmedetomidine could improve allograft function for patients undergoing kidney transplantation. We are planning a single-center randomized controlled trial to confirm the hypothesis, comparing the incidence of DGF between kidney allograft recipients receiving either dexmedetomidine or normal saline, administered during and after surgery for a total of 24 hours. The results of this study will provide evidence to advocate the clinical use of dexmedetomidine in the perioperative period of DCD kidney transplantation.

5. Study Design

5.1 Description of this study

This is a researcher-initiated, randomized, double-blind, placebo-controlled study, with parallel-arms and a superiority design. We hypothesize that the perioperative use of dexmedetomidine for a total 24 hours (at a rate of 0.4 μg/kg/h which is started right after anesthesia induction and lasts throughout the transplant procedure, followed by dexmedetomidine infusion at a rate of 0.1 μg/kg/h after surgery in a designated transplant care unit) will decrease the incidence of DGF after DCD kidney transplantation.

After screening of eligibility, patients who meet the enrollment criteria will be randomized to receive either a dexmedetomidine (intervention group) or a normal saline (placebo control group). The randomization performed at the last available moment, together with the double blindness of the study design, will reduce most potential biases. Both dexmedetomidine and normal saline are provided as clear aqueous solutions in identical bottles and labeled with previous set code. All the patients and the study personnel, including those involved in the transplant care unit, data collection, data entry or data analysis will be blinded to treatment assignment for the duration of the study. Consented patients will receive standard perioperative managements and corresponding study interventions.

After anesthesia induction, patients will receive either dexmedetomidine or normal saline. The hemodynamics of the patients will be tightly monitored. Data will be collected by trained observers who will not participate in patient care and will be blinded to the administered drug. Study outcomes are assessed during the hospital stay and at 30 days after the transplantation procedures.

5.2 Duration of this study

Study team physicians are responsible for screening all patients undergoing a scheduled procedure. The number of eligible, consented, enrolled, and randomized patients is recorded in addition to the reasons for non-participation in the trial. During their hospital stay, patients are closely monitored and all outcomes are recorded. For this reason, no missing endpoints are expected. After discharge, for patients have a scheduled follow-up visit with the cardiac surgeon at 30 days after surgery. The day

before their appointment, the patient is contacted by a member of the study staff to remind them that a member of the team will collect research data during their visit. This is done by a phone call (or email if unreachable by phone). To avoid missing follow-up, a minimum of three calls are attempted by the study team. Calls are made at different times and dates to increase the probability of contacting patients. Phone numbers provided by the patient on the screening day are used. If the study team is still unable to contact the patient despite the several phone calls, a letter is sent to the home address provided by the patient at their initial screening visit. If all attempts do not provide contact with the patient, a member of the study team may call the patient's primary care physician or another healthcare provider to obtain information regarding the patient's condition (e.g., if deceased).

Based on the experience and our pilot data in our institution, as well as the results of previous studies, it is estimated that it will take 12 months to enroll enough patients (which is estimated by sample size calculation; see 10.1) and added with a 30-day follow up, this study will approximately take 13 months.

6. Study Outcomes and Perioperative Data

6.1 Primary outcome

The primary outcome of this study is the incidence of DGF, defined as the need for dialysis during the first week after kidney transplantation.[7]

6.2 Secondary outcomes

- Repeated dialysis, defined as more than one dialysis session during the first post-transplant week;[8]
- In-hospital acute rejection;
- 30-day serum creatinine;
- 30-day serum cystatin C;
- 30-day estimated glomerular filtration rate (eGFR);[31]
- 30-day allograft survival;
- 30-day patient survival.

6.3 Perioperative data

- Graft function-related parameters (serum creatinine, serum cystatin C, creatinine clearance rate, and urine output) before surgery (except creatinine clearance rate and urine output), on postoperative days 1, 2, 3, 5, and 7, and at the time of hospital discharge;
- Arterial blood gas at the end of surgery;
- Visual analogue scale (VAS) scores for pain at 30 min, 24 hours, and 48 hours postoperatively;
- Sufentanil consumption over 24 hours and 48 hours postoperatively;
- Perioperative bradycardia and hypotension events;
- Transplant induction therapy and immunosuppressive medication;
- Duration of surgery, time to extubation, and length of hospital stay.

The creatinine clearance rate is the urine creatinine concentration multiplied by the average urine volume during the collection period divided by the serum creatinine concentration.[32]

7. Selection of Patients

7.1 Inclusion criteria

- Age ≥ 18 years;
- Diagnosed with end-stage renal disease;
- Scheduled for DCD kidney transplant under general anesthesia.

7.2 Exclusion criteria

- Sick sinus syndrome;
- Atrioventricular block;
- A left ventricular ejection fraction < 30%;
- Multi-organ transplantation.

8. Study Treatment

8.1 Randomization and blinding

An independent biostatistician who is not involved in the subsequent study or data management generates a random sequence number by using an online randomization tool (https://www.sealedenvelope.com/randomisation/), with a 1:1 ratio and permuted block sizes of 2 and 4. The randomization process will be performed at the last available moment to overcome possible biases. The allocation concealment will be ensured by using identical opaque sealed envelopes which are stored in a locked room. According to the random codes, an independent anesthesia nurse will prepare the study medications, either dexmedetomidine (Jiangsu Hengrui Medicine Co, Ltd, Jiangsu, China) or normal saline, in identical syringes which are kept in bags labelled with the study numbers only. Both dexmedetomidine and saline are colourless and clear fluids with similar appearance. The participants, anesthesiologists, surgeons, postoperative observers, and other healthcare providers will be thus fully masked to group allocation until the completion of final analysis.

In case of an emergency (e.g., unexpected rapid deterioration in the patient's clinical status), attending anesthesiologists or surgeons could request unmasking of the treatment allocation, or adjust or interrupt drug infusion if necessary. To maintain the overall quality, legitimacy, and integrity of the clinical trial, unblinding of the test drug may occur only in critical circumstances when severe adverse events happen and considered to be related to dexmedetomidine administration. In this circumstance, the PI. fully documents and explains the reasons for unblinding in a report to the Institutional Review Board (IRB).

8.2 Anesthesia management

All patients will receive no premedication. Upon arrival at the operating room, all patients will receive noninvasive cuff blood pressure, electrocardiography, pulse oximetry, and bispectral index (BIS, Aspect Medical Systems, Newton, MA) monitoring. General anesthesia will be induced using propofol (2 mg/kg) and sufentanil (0.4 μg/kg). All patients will be endotracheally intubated with the use of cisatracurium 0.2 mg/kg, and mechanically ventilated with a tidal volume of 6–8 mL/kg, positive end-expiratory pressure of 5–10 cmH_2O, and driving pressure ≤ 15 cmH_2O. The lung recruitment

maneuver will be performed when needed. Peripheral oxygen saturation will be maintained ≥ 95% via the adjustment of the positive end-expiratory pressure and inspired oxygen fraction. The end-tidal carbon dioxide will be maintained at 35–45 mmHg via the adjustment of the respiratory rate and/or tidal volume. Following anesthesia induction, all patients receive an intra-arterial catheter placed in the radial artery and a central venous catheter via the internal jugular vein guided by ultrasonography. Anesthesia will be maintained with 1–3% sevoflurane inhalation, titrated to maintain the BIS values in the range of 40–60. To provide adequate intraoperative analgesia, the attending anesthesiologist can use additional doses of sufentanil, based on hemodynamic changes of the patients. Additional doses of cisatracurium 0.1 mg/kg will be given for intraoperative muscle relaxation. All patients will be covered by a warming blanket and received warm intravenous fluids with the goal of maintaining a nasopharyngeal temperature of 36–37℃. To ensure the consistency and efficiency of this study, all surgical procedures, anesthesia management, and other perioperative care will be provided by the same medical team. After tracheal extubation, patients will be transferred to a designated transplant unit after surgery.

Hypotension event is defined as a mean arterial pressure < 65 mmHg or a ≥ 20% reduction from baseline, and will be treated with intravenous ephedrine 6–10 mg and/or a dopamine infusion. Bradycardia is defined as a heart rate < 50 beats/min, and will be treated with intravenous atropine 0.5 mg. Lactated Ringer's solution is the default fluid for intravenous volume repletion. Fluid management and need for transfusion will be left to the discretion of the attending anesthesiologists. Intravenous ondansetron 4 mg will be given for preventing postoperative nausea and vomiting (PONV). At the end of surgery, a sufentanil-based patient-controlled intravenous analgesia (PCIA) (sufentanil 1 μg/mL in normal saline) will be initiated until 48 hours after surgery, with a background infusion rate of 1 mL/h, a bolus dose of 2 mL, and a lockout time of 10 min. Patients are encouraged to self-administer their PCIA medications for postoperative pain relief.

8.3 Study drug interventions

All patients will be randomly allocated to the intervention group (dexmedetomidine infusion) or the placebo group (normal saline infusion). The intervention period

commences with induction of anesthesia and lasts for 24 hours. Dexmedetomidine (200 μg/2ml, Jiangsu Hengrui Medicine Co, Ltd, Jiangsu, China) or normal saline 2 mL is provided as clear aqueous solutions in the same bottles. Dexmedetomidine is diluted with normal saline to 50 mL before administration. The patients in the dexmedetomidine group will receive a continuous intravenous dexmedetomidine infusion at a rate of 0.4 μg/kg/h, starting immediately after anesthesia induction and continuing throughout the procedure. After tracheal extubation, patients are all transferred to the designated transplant unit and receive postoperative dexmedetomidine infusion at a rate of 0.1 μg/kg/h. The dexmedetomidine infusion will be stopped 24 hours after the initiation of infusion. To avoid the significant hemodynamic fluctuation, no bolus dose of the study drugs will be given. The patients assigned to the normal saline group will receive normal saline infusion, with the same administration fashion as dexmedetomidine.

8.4 Accountability and compliance

This study is designed to see whether perioperative administration of 24-hour dexmedetomidine brings benefits on kidney graft function for patients undergoing DCD kidney transplantation under general anesthesia. The principal investigator will be responsible that this trial is conducted as specified and in accordance with the applicable regulatory requirements. Jiangsu Hengrui Medicine Co, Ltd, Jiangsu, China helps to provide the blinded intervention drugs and placebos, but the pharmaceutical company will not participate in any other parts of the trial. All enrolled patients will receive standard perioperative treatment and the investigational drug will be delivered according to this prespecified standard protocol. The name and dosage of other relevant medications will be recorded in the Case Report Form.

9. Safety Concerns

9.1 Safety profile of dexmedetomidine

Dexmedetomidine was first approved by Food and Drug Administration (FDA) in 1999 as a sedative. Now it is becoming increasingly popular as it promotes a natural, non-rapid-eye-movement sleep, anxiolysis, and analgesia, without concurrent respiratory depression. Hypotension and bradycardia are potential hemodynamic side effects of dexmedetomidine, but most studies found these effects to be self-limiting and clinically benign. Generally, dexmedetomidine has been administered to millions of patients in various surgical procedures every year for at least 20 years, with a good safety profile in the perioperative use and use for the intensive care.

9.2 Hemodynamic responses and monitoring

The administration of a 1 μg/kg bolus dose of dexmedetomidine induces a transient increase in blood pressure and a reflex decrease in heart rate. This effect can be attenuated by a slow infusion over 10 or more minutes. To avoid this effect, in this trial, we omit the bolus dose and start dexmedetomidine infusion after anesthesia induction. More importantly, dexmedetomidine is advised to be carefully administered in patients predisposed to bradycardia or hypotension. In addition, dexmedetomidine is not recommended in hemodynamically unstable patients or in patients with first- or second-degree atrioventricular block, bradycardia (heart rate < 50 bpm), serious cerebrovascular disease, and ejection fraction < 30%. These patients are excluded from the enrollment of this trial.

Dexmedetomidine has been routinely used for decades in hundreds of millions of patients worldwide and there is no additional safety concern under strict clinical monitoring. In this study, all patients will receive the best available treatment. No additional risk for the study subjects is expected during the dexmedetomidine infusion (0.4 μg/kg/h during surgery followed by 0.1 μg/kg/h until 24 hours after initiation of infusion). The administration of dexmedetomidine follows the clinical routine of our institution and the instructions of this drug.

9.3 Discontinuation of intervention and unmasking

The criteria for patient discontinuation from the study are as follows:

1) Voluntary discontinuation by a patient;

2) Exiting the protocol for safety reasons based on the judgement of the clinical or research staff, including

- Acute worsening of hypotension defined as a decrease in MAP of >20 mmHg that is not attributable to other causes (such as hypovolemia, hemorrhage, or sepsis), but is Generally due to worsening of left ventricular failure with ejection fraction < 30%;
- sudden worsening of bradycardia (HR < 50 bpm) and is not response to the β-receptor agonists;
- New onset of atrioventricular block and considered to be worsen under dexmedetomidine administration.

In case of an emergency (e.g., unexpected rapid deterioration in the patient’s clinical status), attending anesthesiologists or surgeons could request unmasking of the treatment allocation, or adjust or interrupt drug infusion if necessary. To maintain the overall quality, legitimacy, and integrity of the clinical trial, unblinding of the test drug may occur only in critical circumstances when severe adverse events happen and considered to be related to dexmedetomidine administration. In this circumstance, the PI. fully documents and explains the reasons for unblinding in a report to the IRB.

10. Statistical Plan

10.1 Sample size estimation

The sample size of this trial is calculated based on previous studies and our pilot data (see 4.4). The incidence of DGF is reported to be 45.1%–55.3% after kidney transplantation from post-cardiac death donations.[33] In our institution, the pilot data shows that 9 out of 20 (45%) DCD kidney transplant recipients who did not receive dexmedetomidine experienced DGF during the first week after kidney transplantation. However, the therapeutic effect of dexmedetomidine on DGF after DCD kidney transplantation is still unknown. We hypothesize that the incidence of DGF would be reduced by 50% after dexmedetomidine treatment. To detect such a difference with a power of 80% at an alpha level of 0.05 and an allocation ratio of 1:1, the sample size is estimated to be 108 patients (54 in each group). Considering that 5% of the subjects might be lost to follow-up or withdraw their informed consent, we finally decide to enroll 114 patients (57 in each group). The sample size calculation is performed using the PASS software (version 11.0.7; NCSS, LCC., Kaysville, UT).

10.2 Statistical analysis

All data analyses will be carried out according to the pre-established analysis plan. A modified intent-to-treat principle will be followed, including any randomized patient who has undergone their kidney transplantation with their primary outcome available. Data will be stored in CRFs electronically, and statistical analyses will be performed using the SPSS software (version 19.0; IBM SPSS, Chicago, IL) and the GraphPad Prism software (version 7.00; GraphPad, San Diego, CA).

Demographic and baseline characteristics will be summarized and presented with the use of descriptive statistics. Categorical variables will be reported as absolute numbers and percentages. Continuous variables will be reported as mean ± standard deviation (SD) or median and interquartile range (IQR), depending on the distribution of data.

For the primary outcome of DGF incidence, the between-group difference will be determined using the Chi-square test. The therapeutic effect will be evaluated using the odds ratio (OR) and 95% confidence intervals (CIs). Furthermore, the Kaplan–Meier plot will be used to depict time-to-event data (i.e., DGF occurrence during the first post-

transplant week) and to show the cumulative effect of dexmedetomidine on DGF. The dexmedetomidine and saline groups will be compared by the log-rank tests, and the therapeutic effect will be evaluated with the use of hazard ratio (HR) and 95% CI. A two-sided $P < 0.05$ indicates a statistically significant difference.

For the secondary outcomes (the need for repeated dialysis in the first post-transplant week, in-hospital acute rejection, and 30-day serum creatinine, serum cystatin C, estimated glomerular filtration rate, allograft survival, and mortality), the between-group differences will be assessed using the independent t test, Mann–Whitney rank-sum test, Chi-square test, or Fisher exact test, as appropriate. The therapeutic effect will be evaluated with the use of mean differences or ORs and 95% CIs. A prespecified multiple testing correction will be performed for the secondary outcomes using the Bonferroni method. Therefore, a P value < 0.007 (i.e., 0.05/7) indicates a statistically significant difference for the secondary outcomes.

For the non-outcome perioperative data, there is no plan for multiple testing correction. In this context, these perioperative data will be shown as point estimates and 95% CIs only, and the readers should not made definite clinical inferences from these data. As patients will be closely monitored and all outcomes will be recorded during their hospital stay, as well as attempts to avoid missing follow-up by various contacting methods, no missing data is expected (see 5.2). As a result, there is no plan for the imputation of missing data. Neither an interim analysis nor a prespecified subgroup analysis is planned.

10.3 Statisticians

Trial statisticians:

Xi-sheng Shan, Xiao-wen Meng, Ke Peng

Independent statistician:

Yao-yu Ying (Department of Epidemiology and Biostatistics, School of Public Health, Medical College of Soochow University, Suzhou, Jiangsu, 215123, China)

11. Ethical Considerations

11.1 Ethical approval and clinical trial registration

This study protocol was approved by the Ethics Committee of The First Affiliated Hospital of Soochow University on June 26, 2019 (No. 2019-092). The written and dated approval signed by the Ethics Committee chairman has been obtained. The study was then registered at the Chinese Clinical Trial Registry on August 28, 2019 (www.chictr.org.cn; ChiCTR1900025493). This study will be conducted in accordance with the principles laid down by the World Medical Assembly and all applicable amendments (Helsinki, 1964) and the ICH guidelines for Good Clinical Practice. This clinical trial will be conducted in compliance with international laws and regulations, and laws and regulations of China, as well as any applicable guidelines.

11.2 Informed consent

The investigator (according to applicable regulatory requirements), or a person designated by the investigator, and under the investigator's responsibility, will fully inform the patient of all pertinent aspects of the clinical trial. All participants will be informed to the most fully extent about this study, in languages and terms they are able to understand. Prior to a patient's participation in the clinical trial, he or she MUST sign the written Informed Consent Form. It will also be made clear to the patient that he or she can withdraw from the study at any time without giving reasons and that they will not be in any way disadvantaged by this. Any Informed Consent will be retained by the Investigator. A copy of the signed and dated written Informed Consent Form will be provided to the patient.

11.3 Responsibilities

The investigators should perform the clinical trial in accordance with this clinical trial protocol, ICH/Good Clinical Practice, and the applicable regulatory requirements. The investigators ensure compliance with all procedures required by the clinical trial protocol and with all study required procedures. The investigator agrees to provide all information requested in the Case Report Forms (CRFs) in an accurate and legible manner.

12. Data Management and Clinical Trial Report

12.1 Source documents

According to the ICH/Good Clinical Practice, the monitoring team must check the Case Report Form (CRFs) entries and the source documents.

12.2 Case Report Forms (CRFs)

It is the responsibility of the investigators to maintain adequate and accurate CRFs records. All CRFs will be completed electronically in their entirety to ensure accurate interpretation of data.

12.3 Data protection

Data will be stored in the electronic database without indicating the name of the patients (a numeric code will be used).

12.4 Clinical trial report

The principal investigator will be responsible for preparing a clinical trial report. When all data have been fully analyzed, the results of the clinical trial will be communicated to all investigators and to the Competent Authority.

13. References

1. United States Renal Data System. 2018 USRDS annual data report: Epidemiology of kidney disease in the United States. National Institutes of Health, National Institute of Diabetes and Digestive and Kidney Diseases 2018;Bethesda, MD.
2. Husain SA, Chiles MC, Lee S, et al. Characteristics and Performance of Unilateral Kidney Transplants from Deceased Donors. Clin J Am Soc Nephrol 2018;13:118-27.
3. Axelrod DA, Schnitzler MA, Xiao H, et al. An economic assessment of contemporary kidney transplant practice. Am J Transplant 2018;18:1168-76.
4. Gaston RS. Improving Long-Term Outcomes in Kidney Transplantation: Towards a New Paradigm of Post-Transplant Care in the United States. Trans Am Clin Climatol Assoc 2016;127:350-61.
5. Huang J, Millis JM, Mao Y, Millis MA, Sang X, Zhong S. A pilot programme of organ donation after cardiac death in China. Lancet 2012;379:862-5.
6. Chinese Society of Organ Transplantation CMA. National guidelines for donation after cardiac death in China. Hepatobiliary Pancreat Dis Int 2013;12:234-8.
7. Kidney Disease: Improving Global Outcomes Transplant Work G. KDIGO clinical practice guideline for the care of kidney transplant recipients. Am J Transplant 2009;9 Suppl 3:S1-155.
8. Schnuelle P, Gottmann U, Hoeger S, et al. Effects of donor pretreatment with dopamine on graft function after kidney transplantation: a randomized controlled trial. JAMA 2009;302:1067-75.
9. Wu WK, Famure O, Li Y, Kim SJ. Delayed graft function and the risk of acute rejection in the modern era of kidney transplantation. Kidney Int 2015;88:851-8.
10. Bahl D, Haddad Z, Datoo A, Qazi YA. Delayed graft function in kidney transplantation. Curr Opin Organ Transplant 2019;24:82-6.
11. Ponticelli C. Ischaemia-reperfusion injury: a major protagonist in kidney transplantation. Nephrol Dial Transplant 2014;29:1134-40.
12. Tedesco-Silva HJ, Mello Offerni JC, Ayres Carneiro V, et al. Randomized Trial of Machine Perfusion Versus Cold Storage in Recipients of Deceased Donor Kidney Transplants With High Incidence of Delayed Graft Function. Transplant Direct 2017;3:e155.

13. Schnuelle P, Schmitt WH, Weiss C, et al. Effects of Dopamine Donor Pretreatment on Graft Survival after Kidney Transplantation: A Randomized Trial. Clin J Am Soc Nephrol 2017;12:493-501.

14. Sureshkumar KK, Hussain SM, Ko TY, Thai NL, Marcus RJ. Effect of high-dose erythropoietin on graft function after kidney transplantation: a randomized, double-blind clinical trial. Clin J Am Soc Nephrol 2012;7:1498-506.

15. Gaber AO, Mulgaonkar S, Kahan BD, et al. YSPSL (rPSGL-Ig) for improvement of early renal allograft function: a double-blind, placebo-controlled, multi-center Phase IIa study. Clin Transplant 2011;25:523-33.

16. Jordan SC, Choi J, Aubert O, et al. A phase I/II, double-blind, placebo-controlled study assessing safety and efficacy of C1 esterase inhibitor for prevention of delayed graft function in deceased donor kidney transplant recipients. Am J Transplant 2018;18:2955-64.

17. Gerlach AT, Murphy CV, Dasta JF. An updated focused review of dexmedetomidine in adults. Ann Pharmacother 2009;43:2064-74.

18. Ebert TJ, Hall JE, Barney JA, Uhrich TD, Colinco MD. The effects of increasing plasma concentrations of dexmedetomidine in humans. Anesthesiology 2000;93:382-94.

19. Fairbanks CA, Stone LS, Kitto KF, Nguyen HO, Posthumus IJ, Wilcox GL. alpha(2C)-Adrenergic receptors mediate spinal analgesia and adrenergic-opioid synergy. J Pharmacol Exp Ther 2002;300:282-90.

20. Wijeysundera DN, Naik JS, Beattie WS. Alpha-2 adrenergic agonists to prevent perioperative cardiovascular complications: a meta-analysis. Am J Med 2003;114:742-52.

21. Wijeysundera DN, Bender JS, Beattie WS. Alpha-2 adrenergic agonists for the prevention of cardiac complications among patients undergoing surgery. Cochrane Database Syst Rev 2009:CD004126.

22. Zhang JJ, Peng K, Zhang J, Meng XW, Ji FH. Dexmedetomidine preconditioning may attenuate myocardial ischemia/reperfusion injury by down-regulating the HMGB1-TLR4-MyD88-NF-small ka, CyrillicB signaling pathway. PLoS One 2017;12:e0172006.

23. Yang YF, Peng K, Liu H, Meng XW, Zhang JJ, Ji FH. Dexmedetomidine preconditioning for myocardial protection in ischaemia-reperfusion injury in rats by

downregulation of the high mobility group box 1-toll-like receptor 4-nuclear factor kappaB signalling pathway. Clin Exp Pharmacol Physiol 2017;44:353-61.

24. Gao JM, Meng XW, Zhang J, et al. Dexmedetomidine Protects Cardiomyocytes against Hypoxia/Reoxygenation Injury by Suppressing TLR4-MyD88-NF-kappaB Signaling. Biomed Res Int 2017;2017:1674613.

25. Farag E, Argalious M, Abd-Elsayed A, Ebrahim Z, Doyle DJ. The use of dexmedetomidine in anesthesia and intensive care: a review. Curr Pharm Des 2012;18:6257-65.

26. Wang K, Wu M, Xu J, et al. Effects of dexmedetomidine on perioperative stress, inflammation, and immune function: systematic review and meta-analysis. Br J Anaesth 2019;123:777-94.

27. Ueki M, Kawasaki T, Habe K, Hamada K, Kawasaki C, Sata T. The effects of dexmedetomidine on inflammatory mediators after cardiopulmonary bypass. Anaesthesia 2014;69:693-700.

28. Cho JS, Shim JK, Soh S, Kim MK, Kwak YL. Perioperative dexmedetomidine reduces the incidence and severity of acute kidney injury following valvular heart surgery. Kidney Int 2016;89:693-700.

29. Yang CL, Chen CH, Tsai PS, Wang TY, Huang CJ. Protective effects of dexmedetomidine-ketamine combination against ventilator-induced lung injury in endotoxemia rats. J Surg Res 2011;167:e273-81.

30. Gu J, Sun P, Zhao H, et al. Dexmedetomidine provides renoprotection against ischemia-reperfusion injury in mice. Crit Care 2011;15:R153.

31. Levey AS, Stevens LA, Schmid CH, et al. A new equation to estimate glomerular filtration rate. Ann Intern Med 2009;150:604-12.

32. Stevens LA, Levey AS. Measured GFR as a confirmatory test for estimated GFR. J Am Soc Nephrol 2009;20:2305-13.

33. Zens TJ, Danobeitia JS, Leverson G, et al. The impact of kidney donor profile index on delayed graft function and transplant outcomes: A single-center analysis. Clin Transplant 2018;32:e13190.
