# Supplementary material for: Effect of Perioperative Dexmedetomidine on Delayed Graft Function Following a Donation-After-Cardiac-Death Kidney Transplant: A Randomized Clinical Trial
Source: JAMA Netw Open. 2022 Jun 3;5(6):e2215217. doi: 10.1001/jamanetworkopen.2022.15217 (PMC9166619; doi:10.1001/jamanetworkopen.2022.15217)
Supplement: Supplement 2. — eMethods. Evolutionary Phases of Organ Transplantation in China, Organ Donation and Procurement, Risk Assessment of Donor’s Kidneys, Anesthetic Care, Perioperative Practice of Dialysis Treatment, Measurements of Creatinine Clearance Rate and Urine Output, and Immunosuppressive Medications eTable. Results of Postoperative Graft Function-Related Parameters eFigure 1. Scheme of Dialysis for Each Patient With Delayed Graft Function eFigure 2. Kaplan-Meier Plot for Delayed Graft Function During 0-7 Days After Kidney Transplantation [file jamanetwopen-e2215217-s002.pdf]

Supplemental Online Content

Shan X, Hu L, Wang Y, Liu H, et al. Effect of perioperative dexmedetomidine on delayed graft function following a donation-after-cardiac-death kidney transplant: a randomized clinical trial. *JAMA Netw Open*. 2022;5(6):e2215217. doi:10.1001/jamanetworkopen.2022.15217

eMethods. Evolutionary Phases of Organ Transplantation in China, Organ Donation and Procurement, Risk Assessment of Donor's Kidneys, Anesthetic Care, Perioperative Practice of Dialysis Treatment, Measurements of Creatinine Clearance Rate and Urine Output, and Immunosuppressive Medications

eTable. Results of Postoperative Graft Function-Related Parameters

eFigure 1. Scheme of Dialysis for Each Patient With Delayed Graft Function

eFigure 2. Kaplan-Meier Plot for Delayed Graft Function During 0-7 Days After Kidney Transplantation

This supplemental material has been provided by the authors to give readers additional information about their work.

© 2022 Shan X et al. *JAMA Network Open.*

eMethods. Evolutionary Phases of Organ Transplantation in China, Organ Donation and Procurement, Risk Assessment of Donor's Kidneys, Anesthetic Care, Perioperative Practice of Dialysis Treatment, Measurements of Creatinine Clearance Rate and Urine Output, and Immunosuppressive Medications

Evolutionary phases of organ transplantation in China

There are three evolutionary phases of organ transplantation in China: the initial exploration phase (1960–2005), the development and construction phase (2005–2015), and the scientific standardization phase (from 2015 to present).[1] The use of organs donated by executed prisoners was stopped on January 1, 2015; since then, voluntary organ donation by citizens has been the only legal source of deceased donor organ transplantation in China.[1-3] The comprehensive China Organ Transplant Response System was established in 2011 to ensure the fairness, transparency, and quality in organ donation and transplantation.[1-3] The number of kidney transplantations increased from 7,040 in 2015 to 13,029 in 2018, with an annual growth rate of approximately 20%.[1]

Organ donation and procurement

In our study, the procedures of organ donation and transplantation conformed to the National Guidelines for Donation after Cardiac Death in China.[4] The fairness and transparency of organ donation and transplantation are safeguarded by the China Organ Transplant Response System.[1,2] All organ donation was voluntary. No kidneys were procured from executed prisoners.

All our donors were controlled donation after cardiac death (DCD) donors (types 3 and 4 in the Maastricht criteria of DCD), also known as donors after brain death followed by cardiac death.[2,5] Apnea test was performed to predict suitability for DCD.[6] Specifically, the patient was disconnected from the ventilator for 10 minutes. If the patient became unstable (oxygen saturation <70% and/or systolic blood pressure <80 mmHg) during the 10-minute observation, the test was aborted. This suggested that cardiac arrest would occur within a short period after extubation and the patient was a suitable candidate for DCD. Potential DCD donors dying beyond 60 minutes after treatment withdrawal were considered unsuitable kidney donors.[7]

Donors' family members were approached by our organ donation coordinators and signed written informed consent. The donated organs were prospectively allocated. The withdrawal of life-sustaining treatment was coordinated by the organ procurement team. In China, there is no legislation for brain death and family members often wish to start the organ donation process after the donor's heart stops beating. Thus, all organ procurement were performed immediately after cardiac death in our study. All donor's kidneys were preserved with cold preservation solution. No machine perfusion pump was used.

Risk assessment of donor's kidneys

The expanded criteria donor was defined as deceased donor >60 years old, or deceased donor aged 50–59 years old with at least two other risk factors (hypertension history, serum creatinine >1.5 mg/dL, or cerebrovascular cause of death).[8] The donor's kidneys were evaluated using the donor-only US kidney donor

© 2022 Shan X et al. *JAMA Network Open.*

risk index (KDRI) and Chinese donor delayed graft function (DGF) risk prediction model.[9-11] The US KDRI scores were divided into quintiles (0.45–0.78, 0.79–0.95, 0.96–1.14, 1.15–1.44, and ≥1.45), with a higher quintile indicating a lower rate of long-term graft survival.[9,10] The Chinese donor DGF risk prediction scores were divided into quartiles (0–9, 10–19, 20–29, and ≥30), with a higher quartile indicating a higher risk of DGF.[11]

Anesthetic care

Patients received no sedation and analgesia in the preoperative area. In the operating room, all patients received a standard monitoring including noninvasive cuff blood pressure, electrocardiography, and pulse oximetry. Bispectral index (BIS, Aspect Medical Systems, Newton, MA) monitoring was used to measure the depth of anesthesia.

General anesthesia was induced using propofol (2 mg/kg), sufentanil (0.4 μg/kg), and cisatracurium (0.2 mg/kg). All patients were endotracheally intubated and mechanically ventilated with a tidal volume of 6–8 mL/kg (predicted body weight), positive end-expiratory pressure of 5–10 cmH_2O, and driving pressure ≤ 15 cmH_2O. The lung recruitment maneuver was applied when clinically indicated. The inspired oxygen fraction was 50%. Pulse oxygen saturation was maintained ≥ 95% via the adjustment of the positive end-expiratory pressure and inspired oxygen fraction. The end-tidal carbon dioxide was maintained at 35–45 mmHg via the adjustment of the respiratory rate and/or tidal volume.

Following anesthesia induction, all patients received an intra-arterial catheter placed in the radial artery at the wrist level for continuous blood pressure monitoring and blood sampling. A central venous catheter was placed in the internal jugular vein, guided by ultrasonography, for intravenous fluid repletion and central venous pressure monitoring. General anesthesia was maintained using sevoflurane inhalation, titrated to maintain the BIS value in the range of 40–60. Additional sufentanil was given for intraoperative analgesia. Additional cisatracurium was given for intraoperative muscle relaxation. All patients were covered by a warming blanket and received warm intravenous fluids with the goal of maintaining a nasopharyngeal temperature of 36–37°C. Perioperative care was provided by the same multi-disciplinary team to ensure consistency and efficiency.

Hypotension, defined as a mean arterial pressure <65 mmHg or a ≥20% reduction from the baseline level, was treated with intravenous ephedrine bolus and/or a dopamine infusion. The mean or median value of the multiple measurements obtained before surgery was used as the patient's baseline blood pressure. Bradycardia, defined as a heart rate <50 beats/min, was treated with intravenous atropine bolus or ephedrine if there was coexisting hypotension. Lactated Ringer's solution was the default fluid for intravenous volume repletion. All patients received intravenous ondansetron (4 mg) for postoperative nausea and vomiting prophylaxis. At the end of the surgery, a sufentanil-based patient-controlled intravenous analgesia (sufentanil 1 μg/mL in normal saline), with a background infusion of 1 mL/h, a bolus dose of 2 mL, and a lockout time of 10 min, was initiated and continued until 48 hours after surgery.

Perioperative practice of dialysis treatment

The perioperative care of kidney transplant recipients was based on the Kidney Disease: Improving Global Outcomes (KDIGO) guideline.[12,13] Before the transplant surgery, our patients typically underwent

© 2022 Shan X et al. *JAMA Network Open.*

hemodialysis 3 times per week or peritoneal dialysis every day. The use of dialysis post-transplant depended on the treating physician's clinical assessment incorporating volume load, urine output, serum potassium, serum creatinine, and creatinine clearance rate. Multiple sessions of dialysis were implemented if there were some urgent issues (such as hyperkalemia, heart failure, hypotension during dialysis, and use of vasopressors). The method of postoperative dialysis in patients with DGF was the same dialysis as that before transplant.

Measurements of creatinine clearance rate and urine output

The creatinine clearance rate was the urine creatinine concentration multiplied by the average urine volume during the collection period divided by the serum creatinine concentration.[14] Urine output was measured hourly through the urinary catheter on postoperative day (POD) 1–3. After the urinary catheter was removed on POD 4, 24-hour urine output was collected and measured.

Immunosuppressive medications

The use of immunosuppressive medications was based on the KDIGO clinical practice guideline for the care of kidney transplant recipients.[12,13] The first-line induction therapy was interleukin 2 receptor antagonist (anti-CD25); in patients at high immunologic risk, the lymphocyte-depleting agent (antithymocyte globulin) was used. The maintenance immunosuppression included the calcineurin inhibitor (tacrolimus or cyclosporine), the antiproliferative agent (mycophenolate mofetil or mycophenolic acid), and corticosteroids (methylprednisolone).

eReferences

1. Shi BY, Liu ZJ, Yu T. Development of the organ donation and transplantation system in China. *Chin Med J (Engl).* 2020;133(7):760-765.
2. Huang J, Millis JM, Mao Y, Millis MA, Sang X, Zhong S. A pilot programme of organ donation after cardiac death in China. *Lancet.* 2012;379(9818):862-865.
3. Sun Q, Gao X, Wang H, Ko DS, Li XC. A new era for organ transplantation in China. *Lancet.* 2014;383(9933):1971-1972.
4. Chinese Society of Organ Transplantation, Chinese Medical Association. National guidelines for donation after cardiac death in China. *Hepatobiliary Pancreat Dis Int.* 2013;12(3):234-238.
5. Sanchez-Fructuoso AI, Prats D, Torrente J, et al. Renal transplantation from non-heart beating donors: a promising alternative to enlarge the donor pool. *J Am Soc Nephrol.* 2000;11(2):350-358.
6. Lewis J, Peltier J, Nelson H, et al. Development of the University of Wisconsin donation After Cardiac Death Evaluation Tool. *Prog Transplant.* 2003;13(4):265-273.
7. Suntharalingam C, Sharples L, Dudley C, Bradley JA, Watson CJ. Time to cardiac death after withdrawal of life-sustaining treatment in potential organ donors. *Am J Transplant.* 2009;9(9):2157-2165.
8. Ethier I, Cho Y, Hawley C, et al. Multicenter registry analysis comparing survival on home hemodialysis and kidney transplant recipients in Australia and New Zealand. *Nephrol Dial Transplant.* 2020.

© 2022 Shan X et al. *JAMA Network Open.*

9. Clayton PA, Dansie K, Sypek MP, et al. External validation of the US and UK kidney donor risk indices for deceased donor kidney transplant survival in the Australian and New Zealand population. *Nephrol Dial Transplant.* 2019;34(12):2127-2131.

10. Rao PS, Schaubel DE, Guidinger MK, et al. A comprehensive risk quantification score for deceased donor kidneys: the kidney donor risk index. *Transplantation.* 2009;88(2):231-236.

11. Xue W, Wang C, Chen J, et al. A prediction model of delayed graft function in deceased donor for renal transplant: a multi-center study from China. *Ren Fail.* 2021;43(1):520-529.

12. Kidney Disease: Improving Global Outcomes Transplant Work Group. KDIGO clinical practice guideline for the care of kidney transplant recipients. *Am J Transplant.* 2009;9 Suppl 3:S1-155.

13. Kasiske BL, Zeier MG, Chapman JR, et al. KDIGO clinical practice guideline for the care of kidney transplant recipients: a summary. *Kidney Int.* 2010;77(4):299-311.

14. Stevens LA, Levey AS. Measured GFR as a confirmatory test for estimated GFR. *J Am Soc Nephrol.* 2009;20(11):2305-2313.

© 2022 Shan X et al. *JAMA Network Open.*

eTable. Results of Postoperative Graft Function-Related Parameters

	**Median (IQR)**			
	**Dexmedetomidine (n = 56)**	**Normal saline (n = 55)**	**Difference (95% CI)**	***P* value**
**Serum creatinine, µmol/L**				
Baseline	944.4 (768.7–1075.0)	828.9 (679.1–1177.0)	115.5 (-67.7 to 169.3)	.42
Postoperative day 1	764.9 (522.9–977.1)	704.2 (526.6–1018.0)	60.7 (-140.3 to 99.6)	.76
Postoperative day 2	474.4 (250.1–836.8)	537.7 (263.5–844.7)	-63.3 (-157.4 to 109.5)	.68
Postoperative day 3	271.6 (155.0–708.0)	381.9 (166.9–725.4)	-110.3 (-142.7 to 58.0)	.53
Postoperative day 5	159.7 (108.8–421.0)	196.9 (101.1–365.9)	-37.2 (-58.3 to 39.5)	.91
Postoperative day 7	132.7 (90.5–233.3)	135.5 (89.0–264.8)	-2.8 (-35.4 to 26.2)	.82
Hospital discharge	97.4 (86.1–117.2)	102.6 (86.6–128.4)	-5.2 (-14.6 to 9.7)	.82
**Serum cystatin C, mg/L**				
Baseline	6.9 (6.2–8.6)	7.1 (5.4–8.1)	-0.2 (-0.4 to 0.9)	.47
Postoperative day 1	4.7 (3.2–6.5)	5.0 (4.1–6.5)	-0.4 (-1.1 to 0.6)	.53
Postoperative day 2	2.8 (2.1–4.6)	3.4 (2.2–4.7)	-0.6 (-0.8 to 0.4)	.52
Postoperative day 3	2.7 (2.0–4.6)	3.3 (2.0–4.3)	-0.6 (-0.7 to 0.5)	.83
Postoperative day 5	2.6 (1.9–4.6)	3.2 (1.8–5.2)	-0.6 (-0.8 to 0.5)	.72
Postoperative day 7	2.3 (1.8–3.5)	2.5 (1.8–4.3)	-0.2 (-0.7 to 0.3)	.45
Hospital discharge	1.7 (1.3–2.3)	1.8 (1.5–2.3)	-0.2 (-0.4 to 0.1)	.28
**Creatinine clearance rate, mL/min**				
Postoperative day 1	9.9 (4.9–21.2)	7.9 (2.0–10.4)	2.0 (0.5 to 6.8)	.02
Postoperative day 2	29.6 (9.7–67.4)	14.6 (3.8–45.1)	15.0 (0.4 to 18.5)	.03
Postoperative day 3	39.7 (12.6–73.6)	24.5 (6.7–69.3)	15.2 (-2.6 to 17.9)	.19
Postoperative day 5	47.4 (20.0–76.5)	39.9 (15.1–74.2)	7.5 (-6.1 to 18.1)	.36
Postoperative day 7	53.0 (32.3–80.3)	48.4 (20.0–69.3)	4.6 (-5.2 to 19.0)	.30
Hospital discharge	67.0 (53.1–82.9)	58.7 (42.7–80.7)	8.3 (-1.3 to 18.6)	.09
**Urine output, mL/h**				
Postoperative day 1	124.6 (48.2–247.6)	88.0 (23.1–186.7)	36.6 (-2.3 to 79.6)	.08
Postoperative day 2	106.5 (66.3–175.6)	82.9 (27.1–141.9)	23.6 (0.2 to 59.0)	.04
Postoperative day 3	99.9 (62.7–152.7)	94.8 (42.7–136.5)	5.1 (-13.6 to 40.0)	.38
Postoperative day 5	125.0 (75.2–163.7)	105.4 (54.6–152.9)	19.6 (-11.2 to 42.1)	.23
Postoperative day 7	126.1 (98.0–151.3)	107.0 (82.5–137.5)	19.1 (1.7 to 36.3)	.03
Hospital discharge	110.4 (92.8–121.9)	97.1 (77.5–113.8)	13.3 (4.2 to 22.5)	.006

Abbreviations: IQR, interquartile range; CI, confidence interval.

© 2022 Shan X et al. *JAMA Network Open.*

eFigure 1. Scheme of Dialysis for Each Patient With Delayed Graft Function

Abbreviations: HD, hemodialysis; PD, peritoneal dialysis; POD, postoperative day.

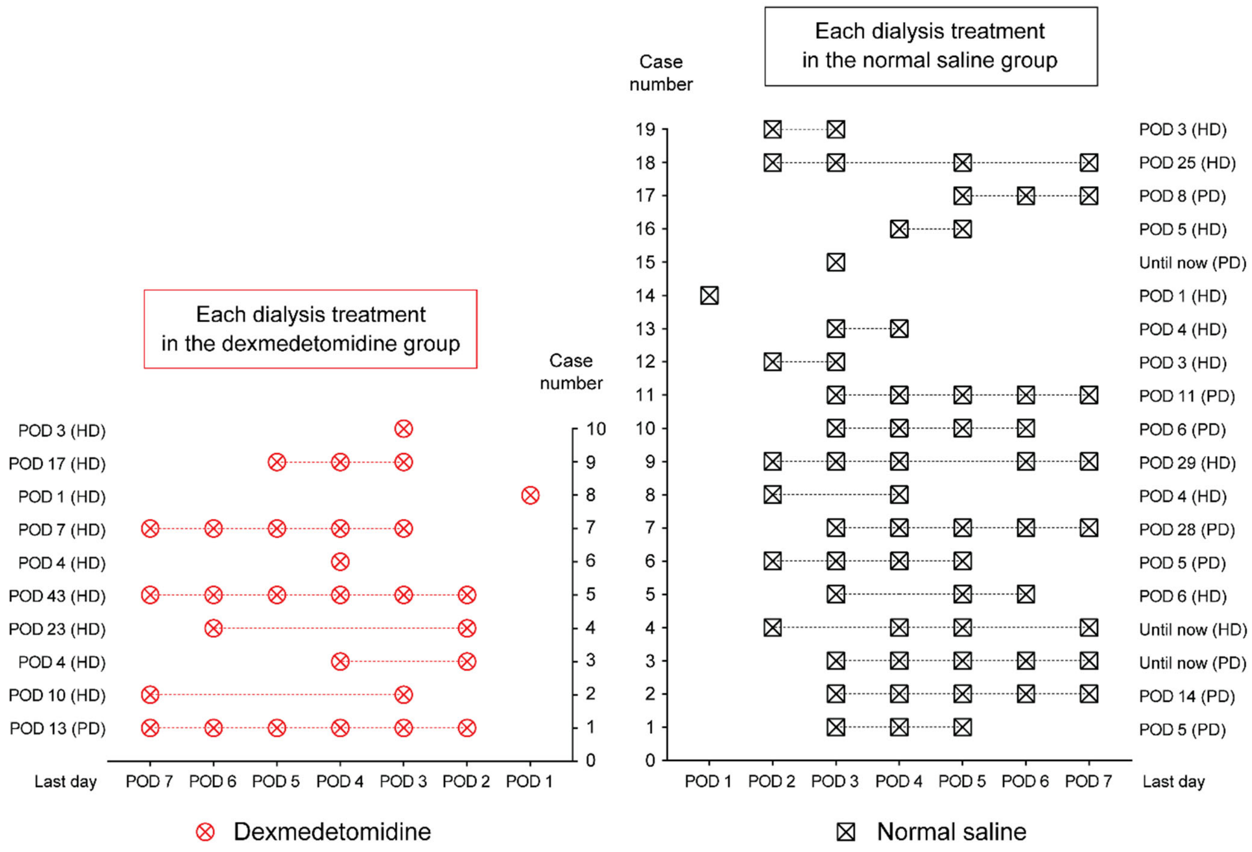

© 2022 Shan X et al. *JAMA Network Open.*

eFigure 2. Kaplan-Meier Plot for Delayed Graft Function During 0-7 Days After Kidney Transplantation

*Log-rank, *P* = .04.
Abbreviations: HR, hazard ratio; CI, confidence interval.

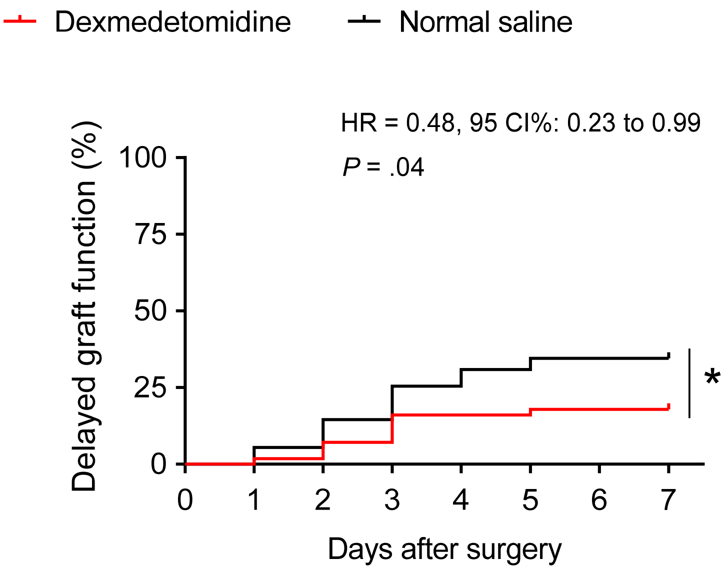

Number at risk

Normal saline	55	52	41	38	36
Dexmedetomidine	56	55	52	47	46

© 2022 Shan X et al. *JAMA Network Open.*
