# Supplementary material for: Effect of Perioperative Dexmedetomidine on Delayed Graft Function Following a Donation-After-Cardiac-Death Kidney Transplant: A Randomized Clinical Trial
Source: JAMA Netw Open. 2022 Jun 3;5(6):e2215217. doi: 10.1001/jamanetworkopen.2022.15217 (PMC9166619; doi:10.1001/jamanetworkopen.2022.15217)
Supplement: Supplement 3. — Data Sharing Statement [file jamanetwopen-e2215217-s00.pdf]

Data Sharing Statement

Shan. Effect of Perioperative Dexmedetomidine on Delayed Graft Function Following a Donation-After-Cardiac-Death Kidney Transplant. *JAMA Netw Open*. Published June 03, 2022. doi:10.1001/jamanetworkopen.2022.15217

Data
Data available: Yes
Data types: Deidentified participant data, Data dictionary
How to access data: The data supporting the results reported in the manuscript can be assessed with approval from the corresponding authors on reasonable request: Fu-hai Ji (jifuhaisuda@163.com) or Ke Peng (pengke0422@163.com).
When available: With publication

Supporting Documents
Document types: None

Additional Information
Who can access the data: anyone requesting the data
Types of analyses: for any purpose
Mechanisms of data availability: with a signed data access agreement
